# Assessment of CuFeSe_2_ ternary nanozymes for multimodal triple negative breast cancer theranostics

**DOI:** 10.1186/s40580-025-00483-4

**Published:** 2025-04-02

**Authors:** Chunmei Yang, Lihong Li, Mingdong Li, Yue Shu, Yiping Luo, Didi Gu, Xin Zhu, Jing Chen, Lu Yang, Jian Shu

**Affiliations:** 1 Department of Radiology, The Affiliated Hospital of Southwest Medical University, Precision Imaging and Intelligent Analysis Key Laboratory of Luzhou, Luzhou, Sichuan 646000 China; 2 Department of Oncology, The Affiliated Hospital of Southwest Medical University, Luzhou, Sichuan 646000 China; 3 The Affiliated Hospital, Southwest Medical University, Luzhou, 646000 China

**Keywords:** CuFeSe_2_ nanosheets, Multimodal imaging, Photothermal therapy, Enzyme catalytic therapy, Chemotherapy

## Abstract

Triple negative breast cancer (TNBC) remains a challenge for clinical diagnosis and therapy due to its poor prognosis and high mortality rate. Hence, new methods to achieve TNBC imaging and imaging-guided TNBC therapy are urgently needed. Currently, the combination of chemotherapy with phototherapy/catalytic therapy has become a promising strategy for cancer treatment. Here, multifunctional CuFeSe_2_ ternary nanozymes (CuFeSe_2_-AMD3100-Gem nanosheets) were prepared as high-performance nanotheranostic agents for imaging-guided synergistic therapy of TNBC in vitro and in vivo. CuFeSe_2_-AMD3100-Gem nanosheets not only exhibited outstanding CXCR4-targeted capability and superior photothermal properties, but also produced exact chemical cytotoxicity through the loading of the chemotherapy drug Gemcitabine. Specifically, the CuFeSe_2_-AMD3100-Gem nanosheets simultaneously possessed peroxidase-like activities capable of converting endogenous H_2_O_2_ to hydroxyl radicals (•OH), which could be significantly enhanced under light irradiation. Furthermore, these nanosheets showed remarkable multimodal imaging ability for magnetic resonance imaging (MRI), computed tomography (CT) and infrared thermography in TNBC tumor-bearing mice (4T1 cells). More importantly, the in vitro and in vivo results verified the significant synergistic anticancer effect of the CuFeSe_2_-AMD3100-Gem nanosheets by combining photothermal therapy and enzyme catalytic therapy with chemotherapy. In conclusion, these advantages demonstrate the powerful potential of CuFeSe_2_ ternary nanozymes for imaging-guided synergistic photothermal/catalytic/chemical therapy for TNBC.

**Graphical Abstract:**

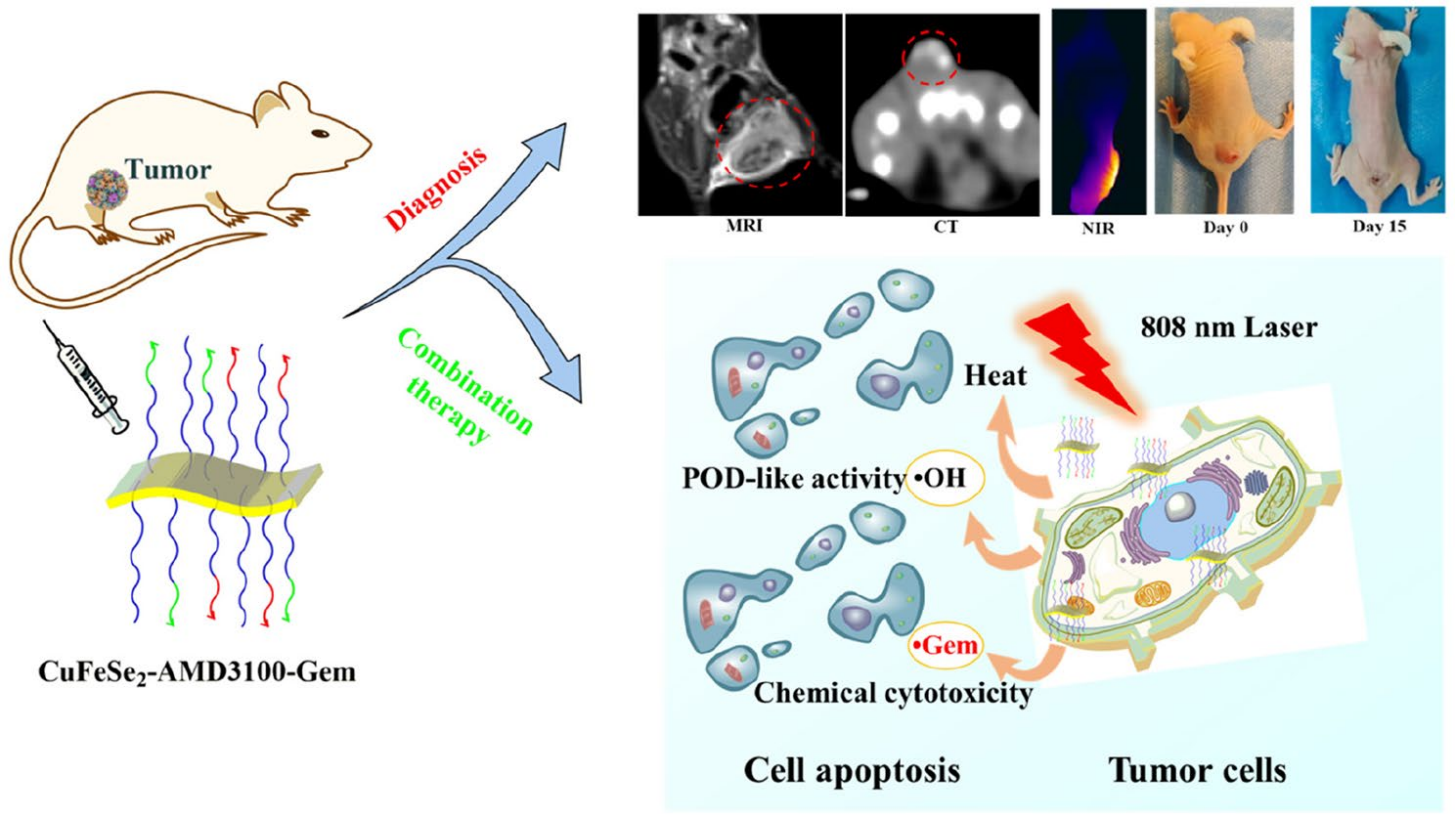

**Supplementary Information:**

The online version contains supplementary material available at 10.1186/s40580-025-00483-4.

## Introduction

Triple negative breast cancer (TNBC) has a poor prognosis and high mortality rate and is the most malignant subtype of breast cancer, seriously threatening women’s life [1, 2]. Less than 30% of patients with TNBC obtain a complete response, and the recurrence and mortality rates remain higher than those of non-TNBC subtypes [3, 4]. The majority of patients are diagnosed in the advanced period because of the carelessness of women concerning self-inspection and physical examination of the breast, which can lead to a low survival rate and a poor prognosis [5, 6]. Currently, multiple imaging techniques have been utilized for the diagnosis of TNBC, e.g., computed tomography (CT) [7], mammography [8], magnetic resonance imaging (MRI) [9], ultrasound [10] and positron emission tomography [11]. Specifically, MRI has been verified as a powerful and sensitive technique for diagnosing TNBC due to its high soft tissue contrast [12, 13]. However, individual imaging has inherent limitations, and it is necessary to use contrast agents for the precise diagnosis of diseases because of the lower sensitivity and clarification of noncontrast techniques. Thus, exploring a.

**Scheme 1:**
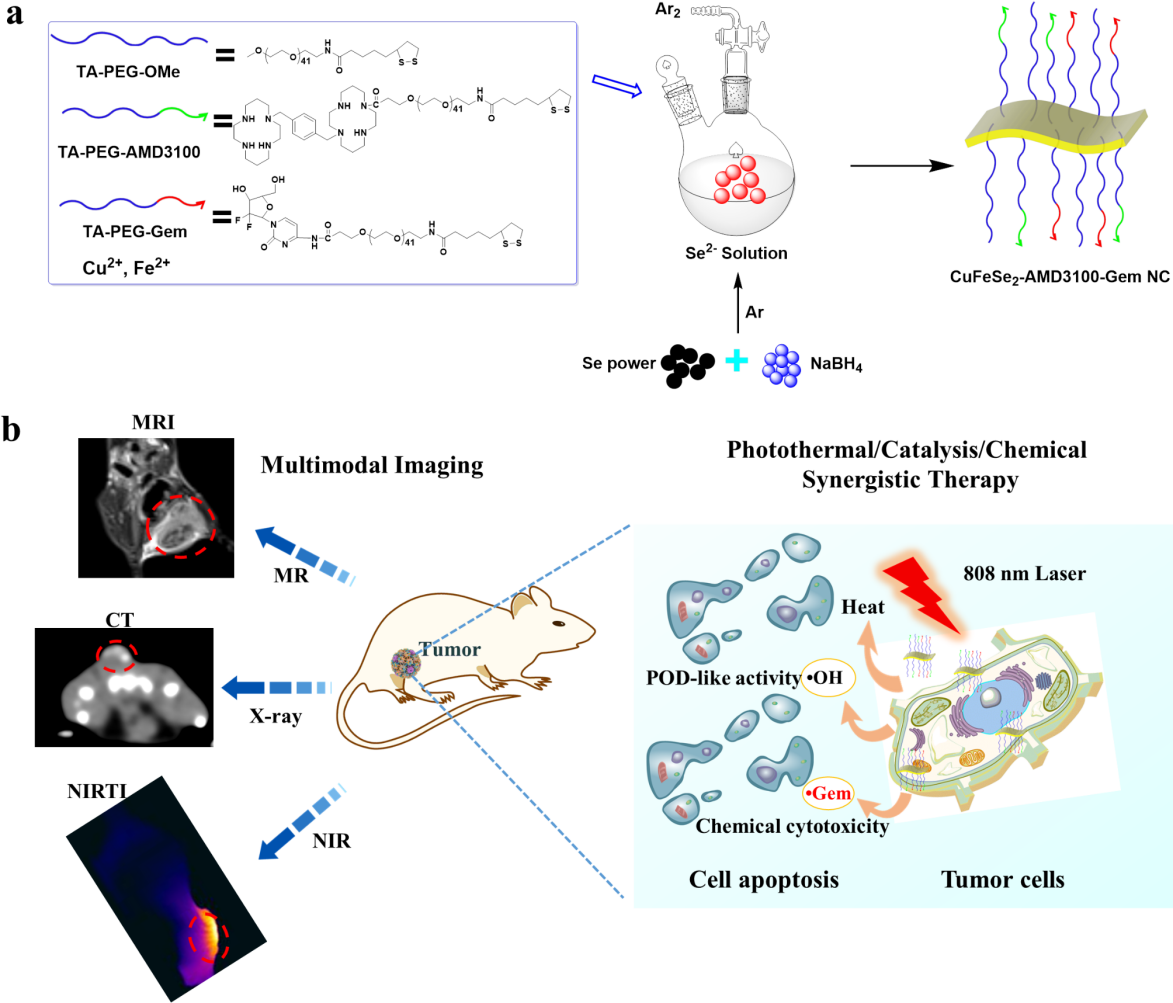
Schematic illustration of the fabrication of CuFeSe_2_-AMD3100-Gem nanosheets for multimodal imaging-guided photothermal/catalytic/chemical synergistic therapy

multimodal imaging contrast agent with high specificity and sensitivity for accurate diagnosis and follow-up monitoring of TNBC is highly important.

At present, the main approaches used to treat TNBC include surgery, chemotherapy, radiation therapy and complementary therapies [14]. Nevertheless, each of these methods has their own drawbacks, and a single treatment modality may not achieve significant therapeutic effects due to tumor aggressiveness and individual differences. Currently, photothermal therapy (PTT), which can achieve thermal ablation of cancer cells through the conversion of light energy into heat energy, has attracted increased interest in medicine and is considered an effective method for cancer therapy [15, 16]. PTT has been used in the thermal therapy of various tumors, such as glioma [17], breast cancer [18], and liver cancer [19]. In the last two decades, many nanoplatforms have been synthesized for PTT, including graphene oxide [20], carbon nanoparticles and nanotubes [21–23], metal nanoparticles of various compositions [24–26], and other inorganic nanoparticles [27, 28]. These nanoplatforms have exhibited promising photothermal properties and may serve as potential photosensitizers for cancer therapy. Currently, nanozyme catalytic therapy has also attracted much attention and has become a new tumor treatment method that induces oxidative stress and kills tumor cells by catalyzing the production of reactive oxygen species (ROS) [29, 30]. An increasing number of nanozymes have been reported for synergistic phototherapy and/or immunotherapy for treating malignancies, which can further enhance therapeutic efficacy and reduce adverse effects during treatment compared with single therapies [34, 31–35].

Cu-Fe-Se, a ternary chalcogenide in the I-III-VI_2_ group, is a promising nanoplatform for multimodal imaging and tumor PTT because of its superior superparamagnetic properties, X-ray attenuation and high photothermal conversion efficiency. To date, some nanoparticles based on Cu-Fe-Se have been synthesized and reported for imaging and/or PTT of tumors, such as CuFeSe_2_-PEG-FA [36], BG-CFS scaffolds [37], CuFeSe_2_@PTMP-PMAA [38], CFS@DOX [39], CFS-S-DOX [40], CuFeSe_2_@MIL-100(Fe)-AIPH [41], CuFeSe_2_-AIPH@BSA [42] and MPEG-PCL@CuFeSe_2_ [43]. These studies demonstrated the outstanding biocompatibility and antitumor efficiency of ternary metal chalcogenides, suggesting that the nanoplatform based on Cu-Fe-Se showed prominent promise for the diagnosis and treatment of cancer. However, spherical particles are clearly observed in most developed nanomaterials based on Cu-Fe-Se, whereas only a few studies on two-dimensional (2D) Cu-Fe-Se nanomaterials for the diagnosis or treatment of tumors have been reported because of the complex synthetic processes of water-soluble 2D nanostructures [39]. Compared with ternary spherical nanoparticles, 2D Cu-Fe-Se nanomaterials exhibit superior loading capacity for anticancer drugs due to their large surface area, negative charged surface and multifunctional groups. Furthermore, 2D nanomaterials’ increased light absorption greatly improves their photothermal conversion efficiency and the ability to generate ROS, which is highly desired in phototherapy of tumor or antibacterial treatment. Hence, 2D nanostructures based on Cu-Fe-Se could be promising theranostic nanoplatforms for multimodal imaging and cancer therapy.

Chemokine receptor 4 (CXCR4) is a G protein-coupled receptor that is overexpressed in breast cancer cells but conserved in normal tissue cells [44]. Therefore, CXCR4 can serve as a specific biomarker for the early detection and treatment of breast cancer. In particular, AMD3100 (Plerixafor) is a small molecule with high binding affinity for CXCR4 that has been reported to be used in various in vivo models and clinical trials [45–47]. At present, conventional anatomical imaging methods lack specificity for the precise diagnosis of TNBC. Therefore, the development of specific molecular probes that target CXCR4 would be highly beneficial for the early diagnosis and targeted therapy of TNBC.

Based on these findings, a multifunctional theranostic nanoplatform was developed based on CuFeSe_2_ for combined imaging and synergistic therapy of TNBC that is capable of guiding clinical strategies and reducing adverse reactions simultaneously (Scheme 1). The prepared multifunctional CuFeSe_2_-AMD3100-Gem nanosheets not only exhibited excellent colloidal stability, CXCR4-targeted capability and biocompatibility, but also possessed superior photothermal properties, peroxidase-like (POD-like) activities and chemical cytotoxicity. Importantly, the unique properties of CuFeSe_2_-AMD3100-Gem nanosheets made them ideally suitable for MRI/CT/Infrared thermal imaging multimodal imaging and synergistic photothermal/catalytic/chemical therapy of TNBC.

## Materials and methods

### Chemicals and materials

Selenium powder, CuCl_2_·2H_2_O, FeSO_4_·7H_2_O and Plerixafor (AMD3100) were purchased commercially from Sigma‒Aldrich, NaBH_4_ was purchased from China National Pharmaceutical Group Beijing Chemical Reagent Co., Ltd. Lipoamide-dPEG_41_-acid (TA-PEG-COOH) and lipoamide-dPEG_41_-m (TA-PEG-OMe) were purchased from ShangHai To YongBio Tech. All other chemicals were obtained from Shanghai Macklin Biochemical Co., Ltd. Roswell Park Memorial Institute-1640 (RPMI-1640) medium, Dulbecco’s minimum essential medium (DMEM), and fetal bovine serum (FBS) were purchased from Gibco.

### Synthesis of the CuFeSe_2_-AMD3100-gem nanosheets

#### Synthesis of TA-PEG-AMD3100

TA-PEG-AMD3100 was synthesized based on a previously reported method [48]. As shown in Scheme S1, TA-PEG-COOH (0. 2 g, 0.1 mmol) and NHS (0.018 g, 0.15 mmol) were dissolved in DCM (5 mL), and the mixture was stirred at 0 °C for 0.5 h. A solution of DCC (0.030 g, 0.15 mmol) in DCM (1 mL) was added dropwise under vigorous stirring. The resulting mixture was stirred for 12 h at room temperature and then filtered and concentrated under vacuum to get TA-PEG-NHS. The product was obtained as a pale white solid (yield = 82%). ^1^H NMR (400 MHz, CDCl_3_) *δ* 3.85–3.76 (m, 4 H), 3.71–3.53 (m, 164 H), 3.50–3.39 (m, 6 H), 3.13 (dd, *J* = 7.1, 5.1 Hz, 4 H), 2.57–2.38 (m, 2 H), 2.20 (t, *J* = 7.4 Hz, 2 H), 2.04–1.85 (m, 2 H), 1.84–1.74 (m, 1H), 1.73–1.60 (m, 4 H), 1.46 (d, *J* = 7.0 Hz, 2 H). ^13^C NMR (101 MHz, CDCl_3_) *δ* 171.86, 168.50, 151.51, 70.73, 70.53, 70.43, 70.14, 68.21, 56.32, 45.73, 40.12, 39.06, 38.35, 36.20, 34.55, 28.82, 25.34, 8.51.

After that, a solution of AMD3100 (0.02 g, 0.04 mmol) in Milli-Q water (3 mL) was added NaHCO_3_ (0.0168 g, 0.2 mmol) to adjust the pH to 7–8, then TA-PEG-NHS (0.04 g, 0.02 mmol) in tetrahydrofuran (3 mL) was added dropwise and the mixture was stirred for 2 days at room temperature. After evaporation of the solvent, the residue was dried under vacuum and washed with diethyl ether to obtain the product as a white solid (yield = 71%). ^1^H NMR (400 MHz, CDCl_3_) *δ* 7.24 (s, 2 H), 7.21 (s, 2 H), 3.80–3.40 (m, 164 H), 3.18–3.02 (m, 5 H), 2.71 (m, 40 H), 2.18–2.13 (m, 2 H), 1.91–1.80 (m, 6 H), 1.72–1.58 (m, 8 H), 1.52–1.37 (m, 5 H). ^13^C NMR (101 MHz, CDCl_3_) *δ* 193.22, 143.00, 137.06, 129.29, 70.40, 70.00, 56.33, 49.41, 48.28, 46.57, 40.11, 39.02, 38.37, 36.96, 35.95, 34.56, 32.62, 31.80, 29.91, 29.57, 29.24, 28.82, 27.84, 26.96, 25.29, 22.57, 19.69, 14.02.

#### Synthesis of TA-PEG-gem

TA-PEG-Gem was also synthesized according to reported procedures [49]. As shown in Scheme S2, TA-PEG-COOH (0.2 g, 0.1 mmol), 2,3,5,6-tetrafluorophenol (0.02 g, 0.12 mmol) and DCC (0.042 g, 0.2 mmol) were dissolved in 2 mL of DMSO, and the mixture was stirred at 90 °C for 12 h to activate the carboxyl groups of TA-PEG_55_-COOH. The resulting precipitate was collected by filtration and used directly without further purification. Then, the activated TA-PEG-COOH was dropwise added into the mixture solution of gemcitabine hydrochloride (30 mg, 0.1 mmol), *N*, *N*-diisopropylethylamine (17 µL, 0.1 mmol) and DMSO (5 mL) under vigorous shaking at 80 °C for 12 h. After being poured into brine and extracted with DCM, the mixture was washed with saturated NaHCO_3_, dried over anhydrous Na_2_SO_4_, filtered and concentrated under vacuum. The product was obtained as a white solid (yield = 66%). ^1^H NMR (400 MHz, CDCl_3_) *δ* 7.73 (s, 1H), 6.69 (s, 1H), 6.53–6.41 (m, 1H), 6.04 (s, 1H), 4.60 (m, 3 H), 4.28–4.11 (m, 2 H), 4.02–3.88 (m, 2 H), 3.84–3.47 (m, 164 H), 3.45–3.34 (m, 4 H), 3.17–3.04 (m, 4 H), 3.00-2.88 (m, 3 H), 2.80 (m, 2 H), 2.51–2.33 (m, 3 H), 2.16 (m, 2 H), 1.95–1.83 (m, 2 H), 1.75 (m, 1H), 1.70–1.58 (m, 4 H), 1.47 (m, 2 H). ^13^C NMR (101 MHz, CDCl_3_) *δ* 184.75, 177.20, 173.01, 70.32, 69.96, 69.84, 66.92, 56.31, 53.88, 40.70, 40.09, 39.01, 38.32, 37.72, 36.06, 34.52, 29.54, 28.78, 28.03, 25.27, 19.21, 17.73, 12.04.

#### Synthesis of CuFeSe_2_-AMD3100-gem nanosheets

The nanosheets were prepared via a minor modified literature protocol [36, 38]. As shown in Scheme 1a, FeSO_4_·7H_2_O (69.5 mg, 0.25 mmol) and TA-PEG ligands (0.4 mM, 1:1:2 molar ratio of TA-PEG-AMD3100, TA-PEG-Gem and TA-PEG-OMe) were mixed in 100 mL of DI water and stirred at room temperature for.

0.5 h. In an argon atmosphere, a mixture of Se (40 mg, 0.5 mmol) and NaBH_4_ (38 mg, 1 mmol) in 100 mL of DI water was stirred at room temperature for 2 h to form a colorless solution, which was added to the above mixture of FeSO_4_ and TA-PEG ligand solutions. Further, CuCl_2_·2H_2_O (43 mg, 0.25 mmol) solution was added to the above mixture under vigorous stirring at room temperature for another 2 h. The prepared nanosheets were precipitated by centrifugation, washed with DI water several times and stored at 4 °C for further application. The CuFeSe_2_-AMD3100 nanosheets were also prepared via a similar method.

### Characterization

Transmission electron microscopy (TEM) and element mapping were conducted on a Tecnai G2 F30 at 300 kV (PEI Ltd., USA) to evaluate the morphology and size characterization of the CuFeSe_2_-AMD3100-Gem nanosheets. Powder X-ray diffraction (XRD) of the CuFeSe_2_-AMD3100-Gem nanosheets was performed via a Bruker D8 Advance diffractometer (Germany) with Cu Kα radiation. The concentrations of Fe, Cu and Se in the CuFeSe_2_-AMD3100-Gem nanosheets were tested via inductively coupled plasma optical emission spectroscopy (ICP‒OES) (Agilent ICPOES730, USA). Fourier transform infrared (FTIR) spectroscopy was performed on a Nicolet 6700 FTIR spectrometer (Thermo Fisher, USA) with KBr. Thermal gravimetric analysis (TGA) was performed on a NETZSCH STA 449 F3/F5 instrument at the rate of 10 °C/min under a nitrogen atmosphere.

### In vitro CT/MRI and colloidal stability of the CuFeSe_2_-AMD3100-gem nanosheets

CuFeSe_2_-AMD3100-Gem solutions with different concentrations of Fe (0, 5.75, 11.5, 23, and 46 µmol/L) were also prepared to obtain solution CT images, which were obtained via spectral CT (IQON Spectral CT, Philips, Netherlands). As a control, spectral CT imaging of iohexol (Yangzijiang Pharmaceutical Group Limited) at the corresponding concentration was also performed. Similarly, in vitro MRI of CuFeSe_2_-AMD3100-Gem samples (0, 0.75, 1.5, 3, and 6 µmol/L) was carried out on 3.0 T MRI (Siemens, Germany). The parameters of CT scanning were as follows: field of view (FOV) 150 × 150 mm, matrix 512 × 512, tube current 100 mAs, tube voltage 120 kV and slice thickness 0.8 mm. The MRI parameters were shown in Table S1.

To evaluate the colloidal stability of CuFeSe_2_-AMD3100-Gem, CuFeSe_2_-AMD3100-Gem nanosheets were dissolved in different media, such as DMEM, RPMI-1640, normal saline, PBS and FBS, for 1, 3 and 7 days at 37 °C respectively.

### In vitro evaluation of entrapment efficiency, loading efficiency and drug release

To assess the Gem entrapment efficiency and loading efficiency of CuFeSe_2_-AMD3100-Gem nanosheets, we first obtained a standard curve (UV absorbance versus Gem concentration) using known concentration of Gem solutions. Then, the prepared CuFeSe_2_-AMD3100-Gem solution was centrifuged (10000 rpm, 10 min) and the absorbance of the supernatant was measured at 268 nm using a UV-vis spectrophotometer. Finally, the entrapment efficiency and loading efficiency were calculated using the following equations: Entrapment efficiency = Amounts of loaded gem/Amounts of total gem × 100%; Loading efficiency = Amounts of loaded Gem/Amounts of CuFeSe_2_-AMD3100-Gem.

Furthermore, the Gem release pattern of these nanosheets was also investigated in 1.2 mL of PBS with different pH (7.2, 5.8) at room temperature. The absorbance of the supernatant was measured at different periods (0, 2, 4, 12, 24, 30 and 48 h) using UV-visible spectroscopy at 268 nm. Next, the percentage of the Gem release was calculated (Release = Amounts of gem in the supernatant/Amounts of loaded gem × 100%).

### Peroxidase-like activity of the CuFeSe_2_-AMD3100-gem nanosheets

3,3’,5,5’-Tetramethylbenzidine (TMB) was used to estimate the peroxidase (POD)-like activity of the CuFeSe_2_-AMD3100-Gem nanosheets in the presence of H_2_O_2_. Specifically, the changes in solution color were photographed, and the absorbance was measured by a UV-vis spectrophotometer for six samples, including TMB + H_2_O_2_, TMB + CuFeSe_2_-AMD3100-Gem, CuFeSe_2_-AMD3100-Gem + H_2_O_2_, and CuFeSe_2_-AMD3100-Gem + TMB + H_2_O_2_ (5, 10 and 20 µg/mL, respectively). To explore the photothermally enhanced POD-like activity, the solution (CuFeSe_2_-AMD3100-Gem + TMB + H_2_O_2_, 20 µg/mL) was irradiated with an 808 nm laser for 5 min (power density: 2 W/cm^2^). The photo was taken and the absorbance was recorded. To quantitatively evaluate the POD-like activity of CuFeSe_2_-AMD3100-Gem nanosheets, a Michaelis-Menten kinetic study was carried out to obtain the Michaelis-Menten constant (*K*_m_) and maximum reaction rate (*V*_max_).

### Intracellular ROS detection

4T1 cells were plated into 6-well plates and incubated for 24 h at 37 °C in 5% CO_2_. These cells were subjected to various treatments, including the control, laser, CuFeSe_2_-AMD3100-Gem (200 µg/mL), and CuFeSe_2_-AMD3100-Gem + laser (808 nm, 10 min, 1.0 W/cm^2^) treatments. After 4 h of incubation, the 4T1 cells were washed 3 times and stained with 2,7-dichlorodihydrofluorescein diacetate (DCFH-DA) for 25 min in the dark. These cells were further washed 3 times and imaged by a live cell workstation. In addition, after administration, the cells were trypsinized and washed 2 times, after which ROS generation was quantified via a flow cytometer.

### Cytotoxicity assessment and in vitro targeted assessment

For cell cytotoxicity, 4T1 cells were plated into 96-well plates and cultured for 24 h at 37 °C in 5% CO_2_. Next, CuFeSe_2_-AMD3100-Gem at various concentrations (0, 6.25, 12.5, 25, 50, 100, and 200 µg/mL) was added to these wells and incubated for 18 h. Cell viability was assessed via the CCK-8 test.

To assess the targeted ability of the as-synthesized CuFeSe_2_-AMD3100-Gem, 6-well plates were incubated with 4T1 cells for 24 h and then treated with different concentrations of CuFeSe_2_-AMD3100-Gem (0, 100, 200, or 400 µg/mL) for 4 h. As a control, the other 4T1 cells were first treated with AMD3100 (4 mg/mL) for 4 h, and subsequently, different concentrations of CuFeSe_2_-AMD3100-Gem (0, 100, 200, or 400 µg/mL) were added. MCF-10 A cells were also used as another control. Afterward, all the cells were washed with normal saline 3 times, and the cell uptake was observed by an inverted fluorescence microscope (IX 73, Olympus, Japan). All the cells were subsequently digested via pancreatin for MRI. MRI scanning was performed via Siemens Prisma 3.0 T MR. T2WI was performed with a 180 × 180 mm FOV, a 1.4 mm slice thickness, a 256 × 256 matrix, 200/96 ms TR/TE, and 4 excitations. Finally, ICP-OES was used to measure the Fe content in the cells.

### Hemolysis assay

All animal studies were performed following the protocols of the Institutional Animal Care and Use Committee of Southwest Medical University (Approval No. 20220808-003). First, blood was collected from the heart of a female Sprague-Dawley (SD) rat. Then, red blood cells (RBCs) were isolated from the serum by centrifugation (6000 rpm) and purified by washing with PBS six times. Next, the diluted RBCs (0.1 mL) were mixed with different concentrations of CuFeSe_2_-AMD3100-Gem solutions (0, 50, 100, 200, 400 µg/mL, 0.9 mL) and cultured at 37 °C for 3 h. PBS was used as a negative control, and ultrapure water was used as a positive control. The mixed solutions were subsequently centrifuged to dislodge the RBCs. A UV spectrophotometer (UV3600, Shimadzu, Japan) was used to measure the absorbance of the supernatant at 541 nm.

### Biodistribution in vivo and biosafety evaluation

First, 4T1 tumor-bearing BALB/C nude mice were subjected to in vivo organ biodistribution evaluation after intravenous injection of CuFeSe_2_-AMD3100-Gem nanosheets (0.25 mmol Fe/kg body weight, 150 µL). At 8 and 24 h post injection, the mice were sacrificed, and the main organs (lung, heart, kidney, liver and spleen) and tumor tissues were dissected and dissolved in aqua regia solution. Finally, the Fe content in different tissues was quantified via ICP-OES. Mice injected with 5% glucose solution were used as controls.

To investigate in vivo biosafety, BALB/C nude mice were intravenously injected with CuFeSe_2_-AMD3100-Gem nanosheets (0.25 mmol Fe/kg body weight, 150 µL) through the tail vein. The mice (*n* = 6) were then sacrificed at 1- and 21- days post injection. In addition, the mice (*n* = 3) injected with glucose solution were used as controls and were sacrificed at 21 days. The obtained blood was tested to determine several liver and kidney function indicators. Finally, hematoxylin and eosin (H&E) staining was carried out to observe the histology and morphology of major organs (lung, heart, kidney, liver and spleen). The body weights of all the mice were measured every 2 days.

### MRI and CT imaging in vivo

The mouse TNBC models were generated via the subcutaneous inoculation of a 200 µL 4T1 cell suspension into the left leg root of female BALB/C nude mice. After approximately 11 days, the tumor-bearing mice were anesthetized via a small animal ventilator and subjected to MRI and CT imaging.

A wrist coil was used to perform MRI scanning of the mice on 3.0 T Prisma MR (Siemens, Germany). T2WI and T2* mapping was performed both before and after intravenous injection of the CuFeSe_2_-AMD3100-Gem nanosheets (0.25 mmol Fe/kg body weight, 150 µL) at 1, 2, 4, 6 and 8 h post injection. For comparison, MRI of the nude mice was also carried out using nontargeted CuFeSe_2_-PEG nanomaterials at an equivalent dose (0.25 mmol Fe/kg body weight, 150 µL). The scanning parameters were shown in Table S2. The T2* values of the tumors were subsequently measured and recorded.

For CT imaging of tumors, 50 µL of CuFeSe_2_-AMD3100-Gem nanosheets (1.0 mmol Cu/kg body weight) were intratumorally injected into 4T1 tumor-bearing nude mice. Clinical iohexol (1.0 mM I/kg body weight, 50 µL) was applied as a control. IQon CT (Philips, Amsterdam, Netherlands) was used to obtain CT images of the tumor-bearing nude mice, and the CT values of the tumors were further measured (CT scanning parameters: matrix 512 × 512, tube current 60 mAs, tube voltage 120 kV, and slice thickness 0.8 mm).

### Photothermal experiments and anticancer effects of CuFeSe_2_-AMD3100-gem in vitro

First, different concentrations of CuFeSe_2_-AMD3100-Gem dispersion (0, 12.5, 25, 50, and 100 µg/mL) were heated for 5 min with an 808 nm laser (1.5 W/cm^2^), and an FLIR thermal imaging camera was used to monitor the real-time temperature. In addition, the CuFeSe_2_-AMD3100-Gem solution (1 mL, 100 µg/mL) was irradiated with an 808 nm laser (1.5 W/cm^2^) for 5 min, after which the laser was turned off, and the mixture was naturally cooled to room temperature. Four cycles of heating/cooling processes were subsequently performed to assess the photothermal cycle stability of the CuFeSe_2_-AMD3100-Gem dispersion. Subsequently, 1 mL of CuFeSe_2_-AMD3100-Gem solution (100 µg/mL) was further irradiated for 5 min (1.0 W/cm^2^ and 2.0 W/cm^2^, respectively).

To explore the cytotoxicity and phototoxicity of CuFeSe_2_-AMD3100-Gem, 4T1 cells were cultured for 24 h and then treated with different concentrations of CuFeSe_2_-AMD3100-Gem (0, 50, 200 and 400 µg/mL) for 2 h. Further, the 4T1 cells were exposed to an 808 nm laser (1.5 W/cm^2^, 10 min) and incubated for another 2 h. After that, the 4T1 cells were washed with normal saline, stained with a LIVE/DEAD™ Cell Imaging Kit and imaged with an inverted fluorescence microscope to discriminate dead cells (red color) and live cells (green color). Furthermore, the CCK-8 test was also performed to evaluate the phototoxicity of the prepared complex to 4T1 cells. 4T1 cells without laser exposure were used as controls.

Moreover, an apoptosis detection kit was used to evaluate the degree of apoptosis induced by the nanosheet-induced synergistic ablation. First, 4T1 cells were treated with PBS, PBS + laser, CuFeSe_2_-AMD3100-Gem (400 µg/mL), or CuFeSe_2_-AMD3100-Gem + laser (1.5 W/cm^2^, 10 min). These cells were subsequently resuspended in binding buffer at a density of 1 × 10^5^ cells/mL. Thereafter, the cells were stained with fluorescein isothiocyanate (FITC)-labeled Annexin V and propidium iodide (PI) for 15 min in the dark. Finally, a flow cytometer (BD, Accuri C6, U.S.A.) was used to detect the percentage of Annexin V + and PI + cells. The apoptosis rate was defined as the sum of the percentage of Annexin V + cells and the percentage of PI + cells.

### In vivo synergistic photothermal/catalysis/chemical therapeutic effects

The back of each female BALB/C nude mouse was subcutaneously injected with 4T1 tumor cells, and the tumor-bearing nude mice were randomly divided into seven groups (*n* = 5 per groups), including the control, laser, Gem, CuFeSe_2_-AMD3100-Gem, CuFeSe_2_-AMD3100, CuFeSe_2_-AMD3100-Gem + laser, and CuFeSe_2_-AMD3100 + laser groups. The tumors in the laser treatment groups were subsequently exposed to the 808 nm laser (2 W/cm^2^, 10 min) after intravenous injection (0.25 mmol Fe/kg body weight, 150 µL) for 8 h. During irradiation, the real-time tumor temperature and thermography pictures were monitored and taken with an FLIR A300 camera. To observe the histological characteristics of the tumors, one tumor-bearing nude mouse from each group was killed 24 h after treatment, and the tumors were removed, stained with H&E and analyzed through immunohistochemical staining with a rabbit anti-Ki67 antibody. The average body weights and relative tumor volumes were recorded every 2 days after the treatment. The tumor volume was calculated as (width^2^×length)/2.

## Results

### Synthesis and characterization of CuFeSe_2_-AMD3100-gem

**Fig. 1:**
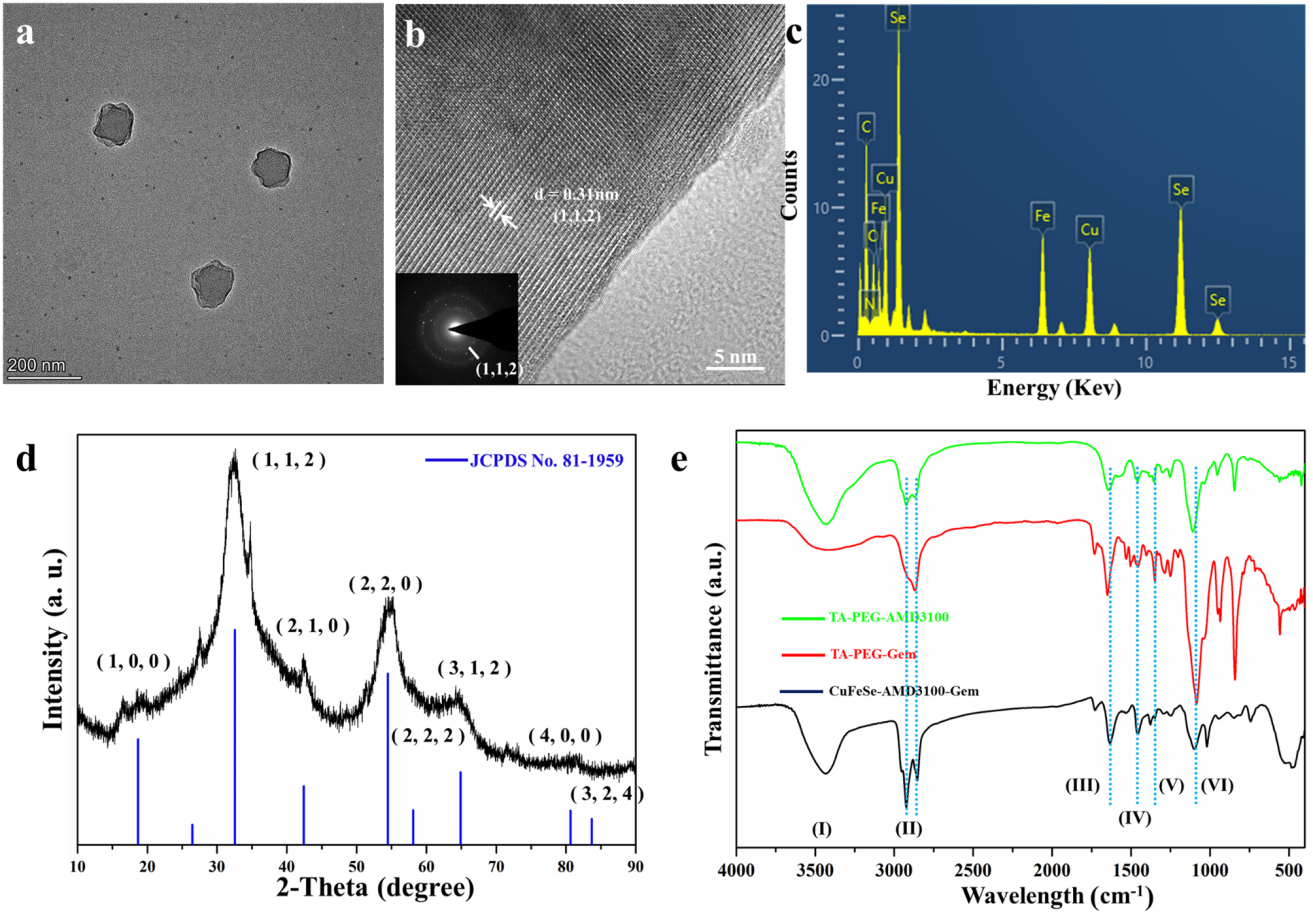
Synthesis and characterization of CuFeSe_2_-AMD3100-Gem nanosheets. (**a**) TEM images of CuFeSe_2_-AMD3100-Gem nanosheets, revealing irregular flaky nanosheets. (**b**) High resolution TEM images and mapping of CuFeSe_2_-AMD3100-Gem nanosheets (inset: SAED pattern). (**c**) EDS spectra of CuFeSe_2_-AMD3100-Gem nanosheets, confirming the presence of Cu, Fe and Se. (**d**) The XRD pattern of CuFeSe_2_-AMD3100-Gem nanosheets and the standard JCPDS (card no. 81-1959) file of CuFeSe_2_. (**e**) FTIR spectra of CuFeSe_2_-AMD3100-Gem nanosheets, TA-PEG-AMD3100 and TA-PEG-Gem (I: O-H stretching; II: C-H stretching; III: C-O stretching; IV: C = C stretching; V: C = O stretching and VI: C-O-C stretching)

The synthesis of CXCR4-targeting two-dimensional CuFeSe_2_-AMD3100-Gem was modified from our previous procedures [36]. The TEM image of CuFeSe_2_-AMD3100-Gem revealed irregular flaky nanosheets with a mean diameter of ~ 119 nm (Fig. 1a and Fig. S1), which may lead to an increase in the surface area/volume ratio, making it possible to load effective drugs for cancer treatment through covalent bonding. The crystal structure of the CuFeSe_2_-AMD3100-Gem nanosheets was tested via high-resolution transmission electron microscopy (HR-TEM). Lattice fringes with an interplanar spacing of 0.31 nm were observed in Fig. 1b, which matched well with the (112) crystallographic plane of tetragonal CuFeSe_2_. The continuous selected area electron diffraction rings in the inset further proved that the interplanar spacings were consistent with those of the (112) planes of the tetragonal structure. In addition, elemental dispersive spectrum analysis revealed the presence of Cu, Fe and Se in the CuFeSe_2_-AMD3100-Gem nanosheets (Fig. 1c). The contents of Cu, Fe and Se in the CuFeSe_2_-AMD3100-Gem nanosheets were quantified via ICP-OES to be 15.57%, 13.82% and 36.25%, respectively, and the ratio of Cu/Fe/Se in the CuFeSe_2_-AMD3100-Gem nanosheets was 1:1:2. Their crystal structure was also further.

characterized by X-ray diffraction (XRD), which fit well with that of CuFeSe_2_ (JCPDS no. 81-1959) (Fig. 1d). Moreover, X-ray photoelectron spectroscopy (XPS) revealed the presence of Cu, Fe and Se in the CuFeSe_2_-AMD3100-Gem nanosheets (Fig. S2). TGA analysis indicated that the amount of CuFeSe_2_ loaded on the CuFeSe_2_-AMD3100-Gem nanosheets was approximately 54.3% (Fig. S3). Furthermore, the FTIR spectra verified the successful surface modification of TA-PEG-AMD3100 and TA-PEG-Gem, and the characteristic peaks at 3428, 2918, 1650, 1456, 1347 and 1081 cm^− 1^ corresponded to O-H stretching, C-H stretching, C-O stretching, C = C stretching, C = O stretching and C-O-C stretching of AMD3100 and Gem, respectively (Fig. 1e). These results confirmed the successful synthesis of the CuFeSe_2_-AMD3100-Gem nanosheets.

**Fig. 2:**
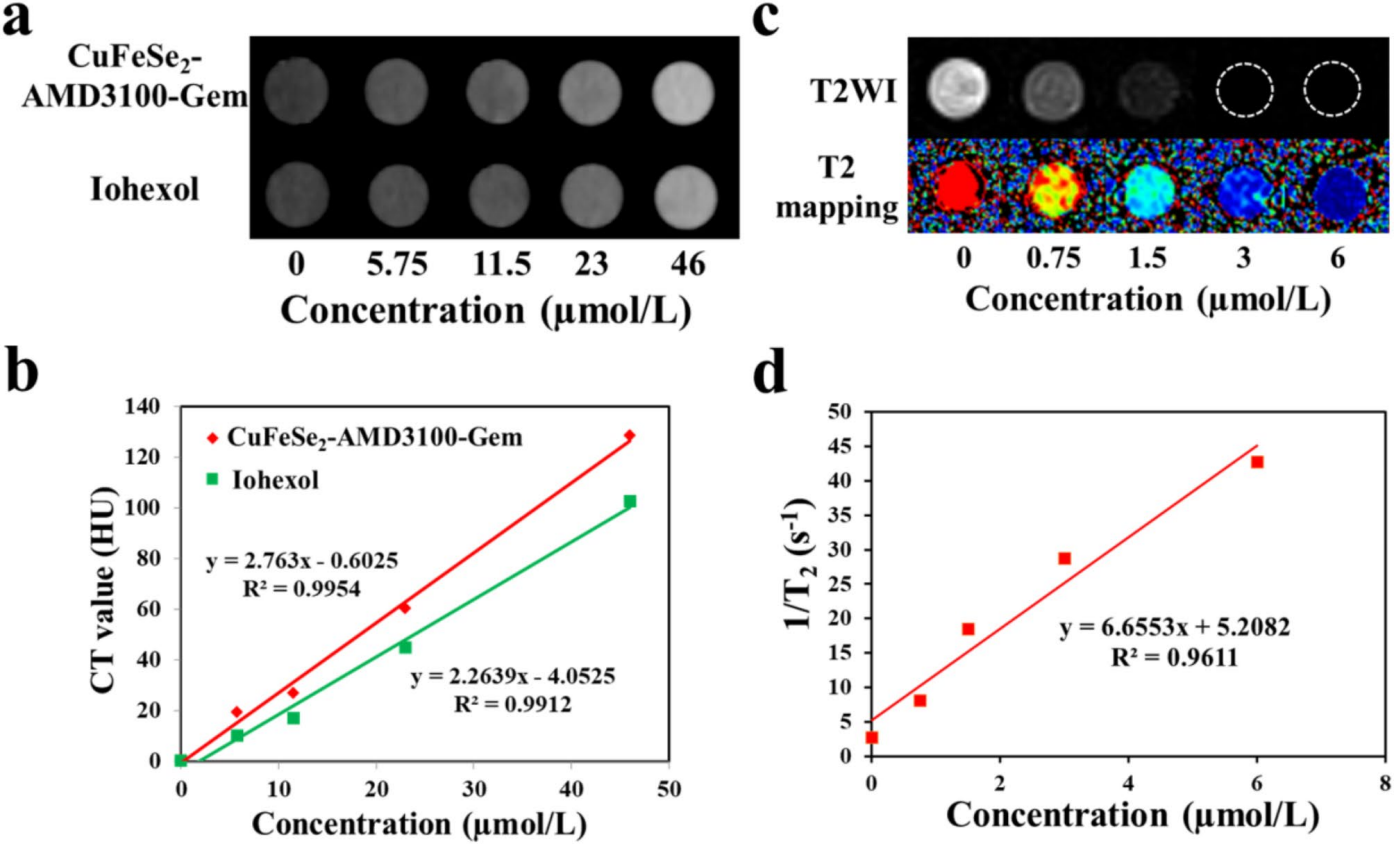
In vitro CT and MRI imaging. (**a**) In vitro CT imaging of CuFeSe_2_-AMD3100-Gem and iohexol with different concentrations. (**b**) The linear correlation between CT values of CuFeSe_2_-AMD3100-Gem (slope: 2.763) and iohexol (slope: 2.2639) with their concentrations respectively. (**c**) T2WI (a strong concentration-dependent darkening effect) and T2 mapping images (a significant color change from red to blue) of CuFeSe_2_-AMD3100-Gem with different concentrations. (**d**) The linear correlation between 1/T_2_ of CuFeSe_2_-AMD3100-Gem solutions with their concentrations (The transverse relativity r_2_ = 6.6553 mM^− 1^s^− 1^)

### In vitro CT/MRI and colloidal stability of CuFeSe_2_-AMD3100-gem

As shown in Fig. 2a and b, the CT signals of CuFeSe_2_-AMD3100-Gem and iohexol increased gradually, and their CT values also increased linearly with increasing concentration. In addition, the CT values of CuFeSe_2_-AMD3100-Gem were higher than those of iohexol at equivalent concentrations, indicating the excellent CT imaging efficacy of our complex over iohexol. For the magnetic properties of CuFeSe_2_-AMD3100-Gem, the T2-weighted image (T2WI) showed a strong concentration-dependent enhancement effect (darkening) with a transverse relativity value (r_2_) of 6.6553 mM^− 1^s^− 1^ (Fig. 2c and d). In particular, the CuFeSe_2_-AMD3100-Gem solution caused a significant decrease in the T2 signal with increasing Fe concentration. These results demonstrated that the CuFeSe_2_-AMD3100-Gem nanosheets have robust potential as contrast agents for CT/MRI dual-modality imaging.

The colloidal stability test revealed that no sediments or aggregates were observed in different media (DMEM, RPMI-1640, normal saline, PBS or FBS) after 1, 3 and 7 days of storage (Fig. S4), which indicated that CuFeSe_2_-AMD3100-Gem had excellent solubility and stability.

### In vitro evaluation of entrapment efficiency, loading efficiency and drug release

The Gem entrapment efficiency, loading efficiency and drug release of CuFeSe_2_-AMD3100-Gem nanosheets were determined.

**Fig. 3:**
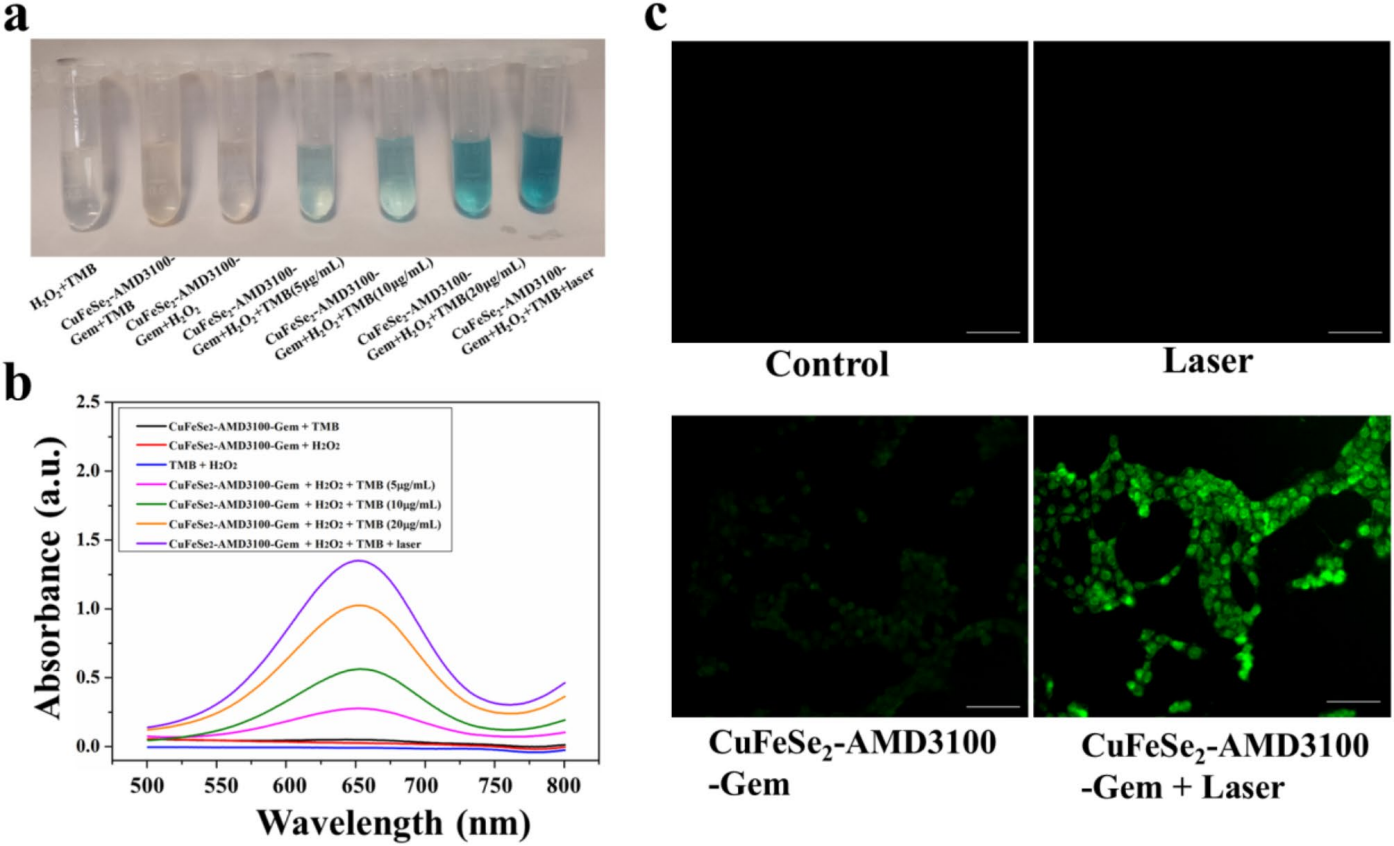
(**a**) and (**b**) POD-like activities of CuFeSe_2_-AMD3100-Gem nanosheets. (**c**) The fluorescence images of 4T1 cells stained by DCFH-DA. Scale bar = 100 μm

using UV-visible spectroscopy at the wavelength of 268 nm. The results indicated that the Gem entrapment efficiency of these nanosheets was about 82.08% and the loading efficiency was about 7.9%. In addition, the Gem release profile of CuFeSe_2_-AMD3100-Gem was investigated in the PBS with different pH for 48 h. As shown in Fig. S5, a slight release pattern was observed within 48 h at pH 7.2. However, Gem release increased gradually with the increasing time at pH 5.8 and there existed significant differences between the amounts of released Gem at normal pH and acid pH, suggesting that an acid environment contributed to the release of Gem. Notably, about 11.7% of Gem was released from CuFeSe_2_-AMD3100-Gem nanosheets after 48 h at pH 5.8, confirming the excellent sustained-release capability of these nanosheets.

### Peroxidase-like activity assay of CuFeSe_2_-AMD3100-Gem nanosheets

In our study, we evaluated the POD-like activity of CuFeSe_2_-AMD3100-Gem via TMB. As shown in Fig. 3a, no blue product was observed in the TMB + H_2_O_2_, TMB + CuFeSe_2_-AMD3100-Gem and CuFeSe_2_-AMD3100-Gem + H_2_O_2_ groups. However, the specific blue color of the oxidized TMB appeared in the CuFeSe_2_-AMD3100-Gem + TMB + H_2_O_2_ (5, 10, 20 µg/mL) groups, and the color gradually intensified as the concentration increased. Amazingly, the blue color of the solution became more obvious after 808 nm laser irradiation, indicating the photothermal-enhanced POD-like activity of CuFeSe_2_-AMD3100-Gem. Furthermore, the absorption changes at 652 nm further demonstrated that the CuFeSe_2_-AMD3100-Gem + TMB + H_2_O_2_ (5, 10, 20 µg/mL) groups presented apparent characteristic absorption peaks compared with those of the TMB + H_2_O_2_, TMB.

**Fig. 4:**
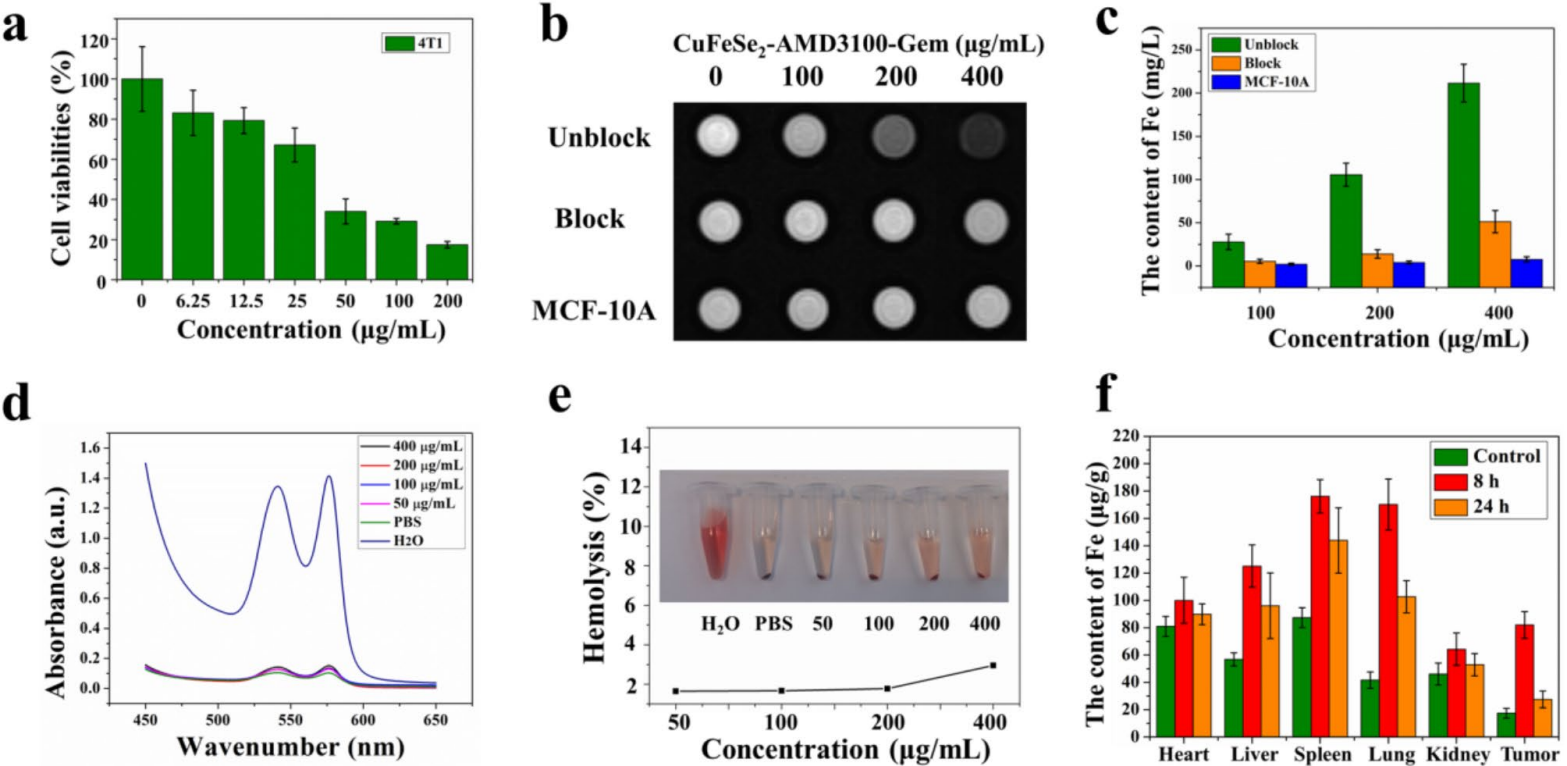
(**a**) Cell viabilities of 4T1 cells after incubation with different concentrations of CuFeSe_2_-AMD3100-Gem nanosheets for 18 h. (**b**) T2-weighted imaging and (**c**) The ICP-OES results of different groups (AMD3100-blocked and unblocked 4T1 cells, MCF-10 A) treated with different concentrations of CuFeSe_2_-AMD3100-Gem. (**d**) UV-vis absorption spectra of the supernatant of CuFeSe_2_-AMD3100-Gem and RBC mixtures. (**e**) Hemolysis of different concentrations of CuFeSe_2_-AMD3100-Gem. (**f**) In vivo organ biodistribution after the intravenous injection of CuFeSe_2_-AMD3100-Gem nanosheets (0.25 mmol Fe/kg body weight, 150 µL) for 8 h and 24 h

+ CuFeSe_2_-AMD3100-Gem and CuFeSe_2_-AMD3100-Gem + H_2_O_2_ groups, suggesting the catalytic activity of these nanosheets (Fig. 3b). More significantly, the absorbance intensity after laser irradiation was greater than that in the other treatment groups, confirming that the POD-like activity could be enhanced by the photothermal effect. Besides, a Michaelis-Menten kinetic study was performed to quantitatively assess the POD-like activity of these nanosheets. *K*_m_ and *V*_max_ were determined to 2.0746 mM and 1.0694 M/s respectively when changing the concentration of the substrate H_2_O_2_ (Fig. S6), suggesting that the remarkable POD-like activity of CuFeSe_2_-AMD3100-Gem nanosheets.

### Intracellular ROS detection

The production of intracellular ROS was explored via the use of DCFH-DA. As presented in Fig. 3c, the CuFeSe_2_-AMD3100-Gem group exhibited green fluorescence compared with the laser and control groups, confirming the ROS production and the POD-like activity of these nanosheets. In addition, stronger fluorescence was observed in the CuFeSe_2_-AMD3100-Gem + laser group, indicating that the production of •OH could be strengthened by the photothermal effect. Flow cytometry analysis revealed that the mean fluorescence intensity of the CuFeSe_2_-AMD3100-Gem + laser group was stronger than that of the other groups (Fig. S7). In other words, CuFeSe_2_-AMD3100-Gem has strong potential to increase intracellular ROS production, and its excellent enzyme-like activity is enhanced by the photothermal effect.

**Fig. 5:**
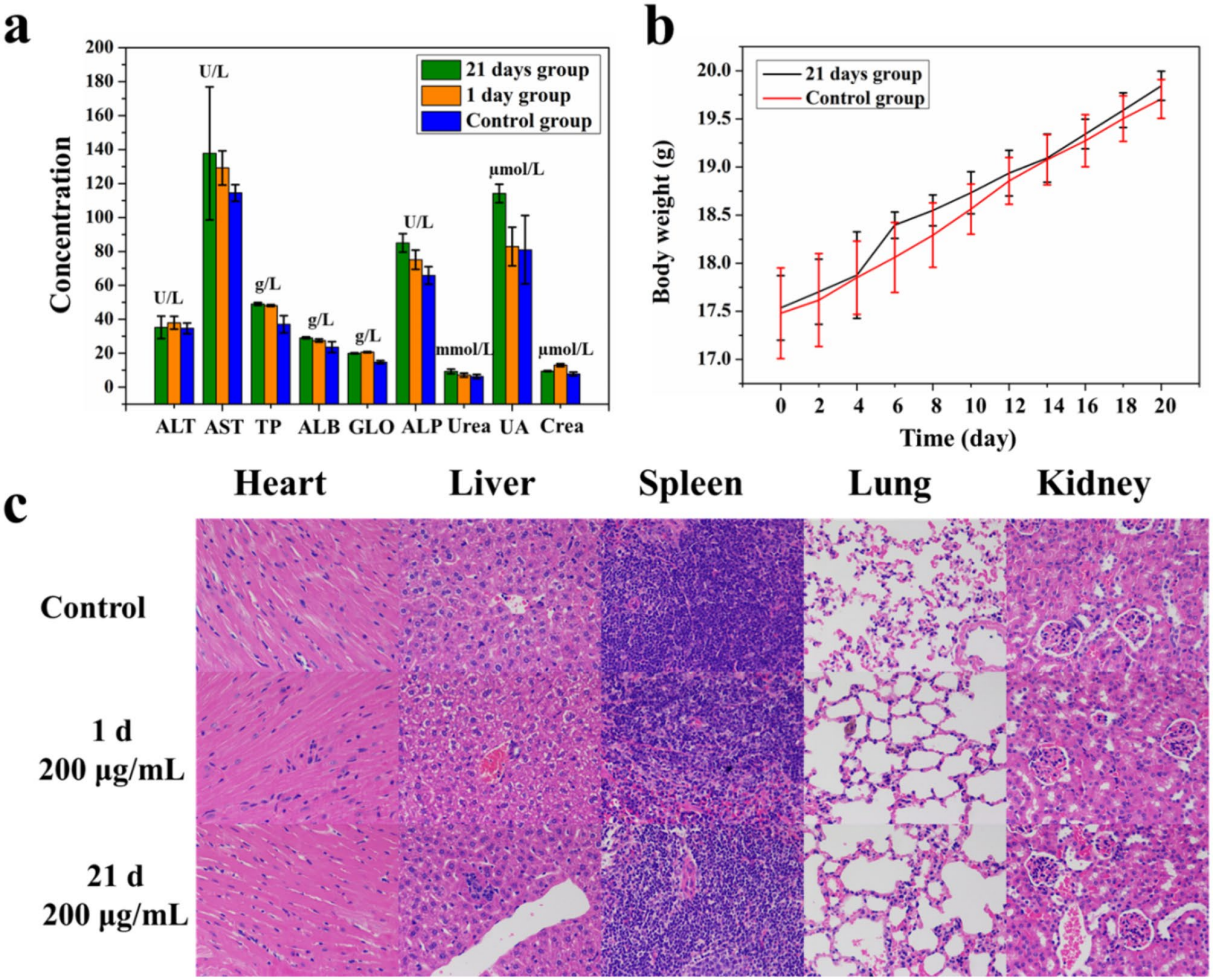
(**a**) The major biochemical markers of mice at 1 and 21 days after intravenous injection of CuFeSe_2_-AMD3100-Gem nanosheets (0.25 mmol Fe/kg body weight, 150 µL). (**b**) The changes in body weight in different treatment groups measured every 2 days. (**c**) H&E staining of major organs for normal mice at different time points after the injection of CuFeSe_2_-AMD3100-Gem nanosheets and 5% glucose solution

### Cytotoxicity assessment and vitro targeted assessment

In the current study, the CCK-8 test was used to assess the cytotoxicity of CuFeSe_2_-AMD3100-Gem. As shown in Fig. 4a, 4T1 cell viability decreased gradually with increasing concentration, and the CuFeSe_2_-AMD3100-Gem nanosheets exhibited significantly increased toxicity toward 4T1 cells whose viability was lower than 80% in the range of 50–200 µg/mL CuFeSe_2_-AMD3100-Gem due to the chemical toxicity of the loaded gemcitabine.

Furthermore, a receptor-blocking assay was carried out to verify the ability of CuFeSe_2_-AMD3100-Gem to target 4T1 cells. The unblocked cells gradually darkened with increasing concentrations of CuFeSe_2_-AMD3100-Gem from T2WI (Fig. 4b). Nevertheless, the MR signal intensities of the block and MCF-10 A groups only slightly decreased at a concentration of 400 µg/mL. Moreover, after these cells were incubated for 4 h with CuFeSe_2_-AMD3100-Gem solution, it was found that more nanosheets could bind to the surfaces of unblocked 4T1 cells than to those of the block and MCF-10 A groups by inverted fluorescence microscopy (Fig. S8), which verified the successful modification of AMD3100. The ICP‒OES results further demonstrated that unblocked 4T1 cells internalized more CuFeSe_2_-AMD3100-Gem nanosheets than blocked cells and MCF-10 A cells at the same concentration (Fig. 4c). These results confirmed that the CuFeSe_2_-AMD3100-Gem nanosheets had outstanding ability to target 4T1 cells, which could contribute to the diagnosis and treatment of 4T1 tumors.

**Fig. 6:**
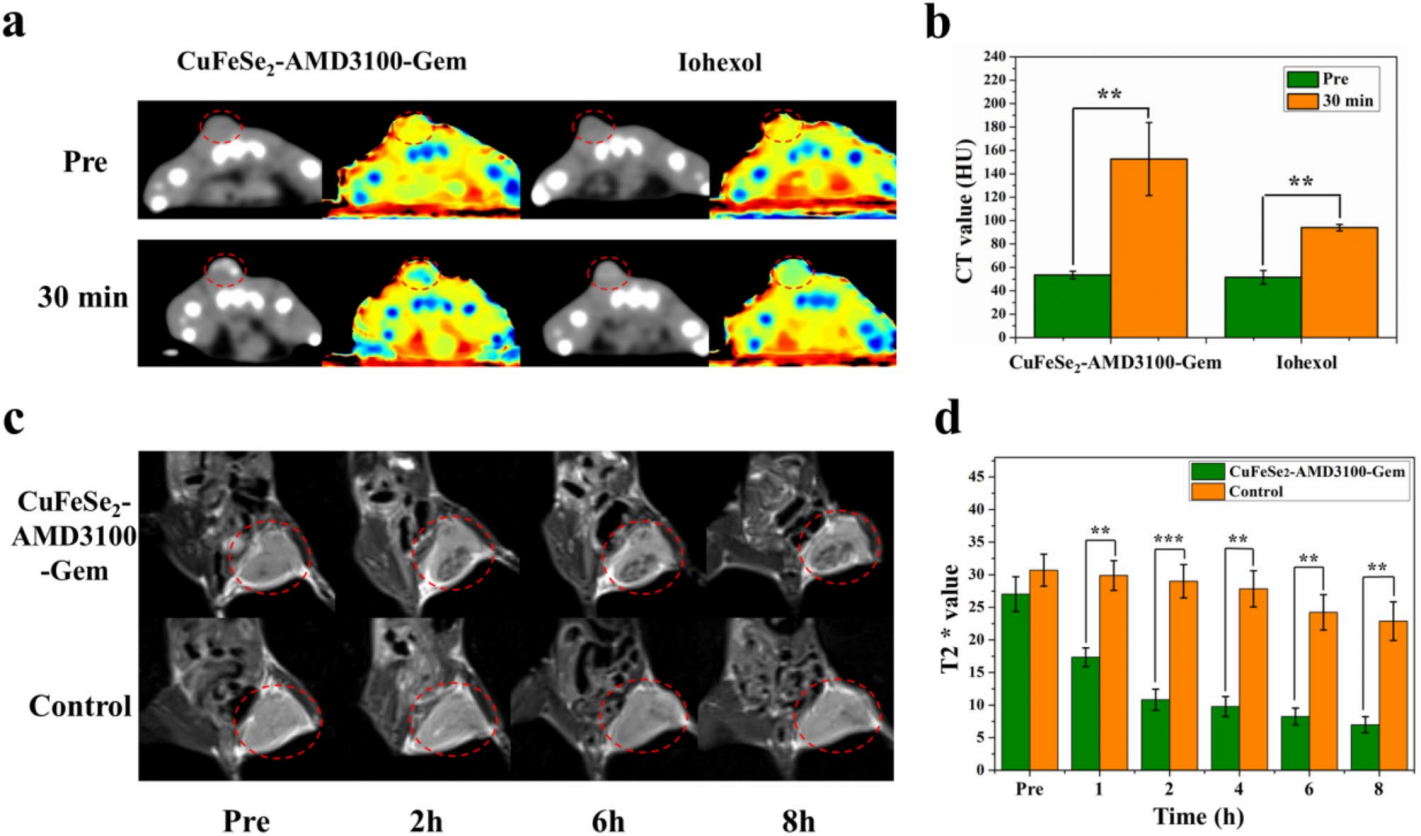
(**a**) CT images of 4T1 tumor-bearing nude mice before and after intratumoral administration of CuFeSe_2_-AMD3100-Gem nanosheets. (**b**) CT values of tumor before and after the injection. (**c**) T2WI of 4T1 tumor-bearing nude mice before and after intravenous injection of CuFeSe_2_-AMD3100-Gem nanosheets at different time points. (**d**) The T2* value of tumor at different time points before and after the injection. The tumor is marked in the red circle

### Hemolysis assay

The hemocompatibility of the CuFeSe_2_-AMD3100-Gem nanosheets was assessed via a hemolysis assay. The results revealed that CuFeSe_2_-AMD3100-Gem at the studied concentrations exhibited admirable hemocompatibility with negligible hemolysis (Fig. 4d). All hemolysis percentages of CuFeSe_2_-AMD3100-Gem were less than 5% in the performed concentration range (50–400 µg/mL) (Fig. 4e), further confirming their good hemocompatibility.

### Biosafety evaluation and biodistribution in vivo

In this study, ICP-OES was used to evaluate the biodistribution of CuFeSe_2_-AMD3100-Gem in major organs and tumors. The results confirmed that the levels of Fe in the lung, heart, liver, spleen and tumor were much greater than those in the control at 8 h postinjection (Fig. 4f). Moreover, the injected CuFeSe_2_-AMD3100-Gem could be cleared from the kidney and tumor tissue, but a portion of the nanosheets enriched in the heart, liver, spleen and lung remained after intravenous administration for 24 h due to of their prolonged blood circulation time.

After in vivo biodistribution evaluation, biochemical detection and histopathological analysis were further employed to assess the in vivo biosafety of the CuFeSe_2_-AMD3100-Gem nanosheets. As shown in Fig. 5a, the major liver function markers (total protein, TP; globulin, GLO; aspartate aminotransferase, AST; albumin, ALB; alkaline phosphatase, ALP; alanine aminotransferase, ALT) and kidney function markers (urine acid, UA; creatinine, Crea; Urea nitrogen, Urea) at the 1st and 21st days post injection were not obviously different from those in the control group, indicating that the CuFeSe_2_-AMD3100-Gem nanosheets had no significant effect on liver or kidney injury. In.

addition, the body weights of the mice increased, and no deaths were observed among the different treatment groups (Fig. 5b). H&E staining verified that there were no obvious abnormalities (e.g., inflammation, hemorrhage, or necrosis) in the major organs of the plants in the CuFeSe_2_-AMD3100-Gem groups compared with those in the control group (Fig. 5c). The above results demonstrated the low toxicity of our CuFeSe_2_-AMD3100-Gem nanosheets.

### MRI and CT imaging in vivo

First, CT imaging of intratumoral injection was performed to evaluate the contrast effect of CuFeSe_2_-AMD3100-Gem. Figure 6a shows that the conventional CT intensity of tumors after injection of CuFeSe_2_-AMD3100-Gem increased and was greater than that of the iohexol group. The corresponding effective atomic number images also displayed a significant pseudocolor transformation before and after intratumoral management. Quantitative analysis further revealed a significant difference in the CT values of the tumors before and after injection, and the CT value of the tumors in the CuFeSe_2_-AMD3100-Gem group was greater than that in the iohexol group at 30 min post injection (Fig. 6b).

Additionally, T2WI and T2* mapping of the nude mice was performed to verify the in vivo MRI feasibility of CuFeSe_2_-AMD3100-Gem. Figure 6c shows the T2W images of 4T1 tumor-bearing nude mice before injection and 2 h, 6 h and 8 h after injection. The MR signal of the tumor gradually decreased over time after intravenous injection of CuFeSe_2_-AMD3100-Gem, and the T2* values of the tumor also decreased with increasing time after injection (Fig. 6d). At approximately 8 h postinjection, the MR signal of the tumor region became the darkest, suggesting that it achieved the best enhancement effect. In the control group, no obvious signal decreased in the tumor region over time, and there was no significant difference in the T2* values of the tumor tissue before and after injection. These results demonstrated that CuFeSe_2_-AMD3100-Gem could serve as an efficient T2-negative contrast agent for the diagnosis of tumors. In summary, these results highlight the potential of CuFeSe_2_-AMD3100-Gem for MRI and CT dual-modality imaging, which could significantly improve the diagnostic sensitivity and accuracy of tumors.

### Photothermal experiments and anticancer effects of CuFeSe_2_-AMD3100-gem in vitro

**Fig. 7:**
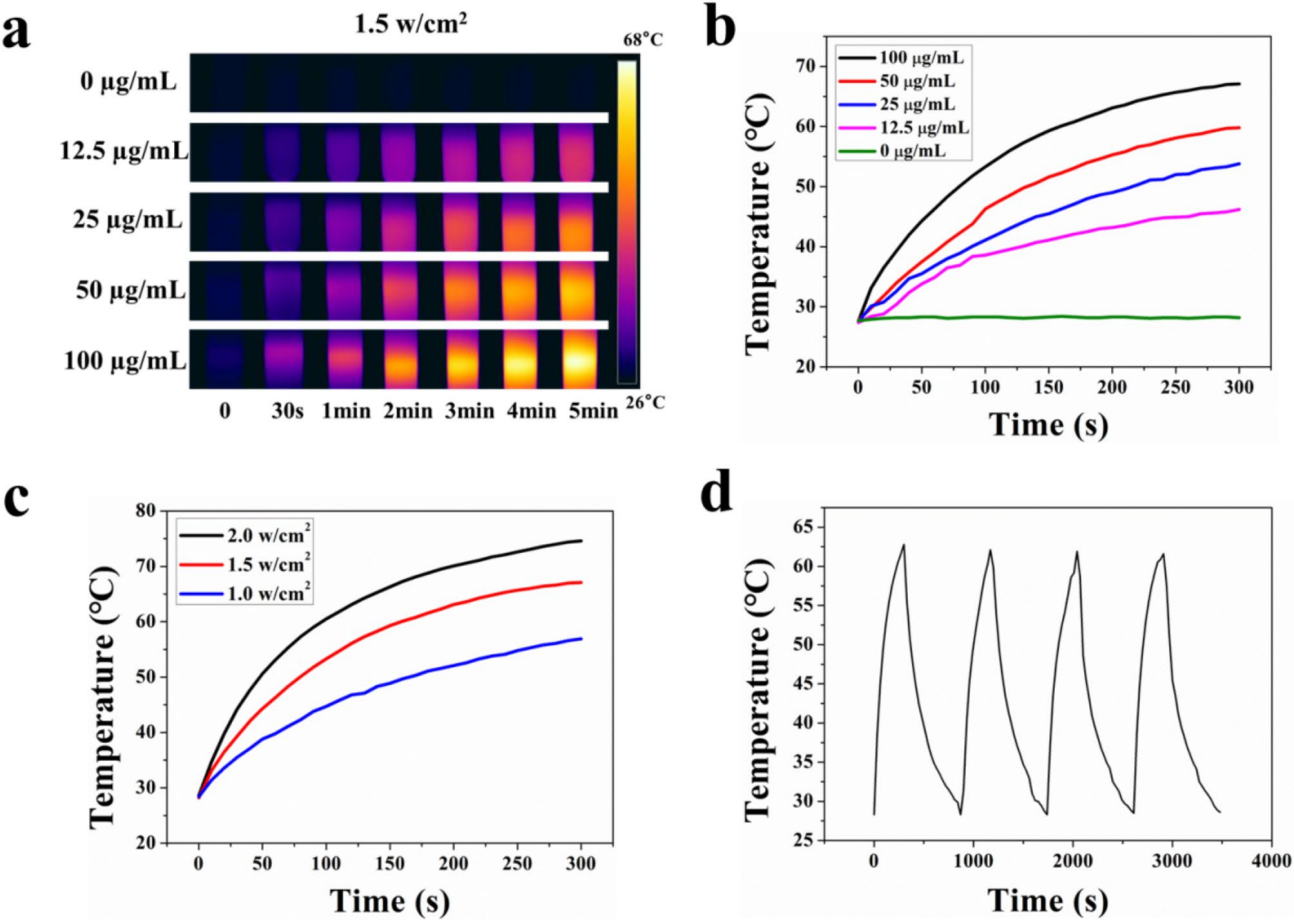
(**a**) Photothermal images of CuFeSe_2_-AMD3100-Gem nanosheets with different concentrations. (**b**) Temperature change curves of different nanosheets concentrations upon irradiation (808 nm, 1.5 W/cm^2^, 5 min). (**c**) Temperature change curves of CuFeSe_2_-AMD3100-Gem nanosheets at various laser power densities (808 nm, 5 min). (**d**) Temperature change curves of CuFeSe_2_-AMD3100-Gem solution during repetitive irradiation (4 times) with laser-on for 5 min and then laser-off

To validate the photothermal performance of CuFeSe_2_-AMD3100-Gem, CuFeSe_2_-AMD3100-Gem solutions with different concentrations were irradiated with an 808 nm laser (1.5 W/cm^2^) for 5 min (Fig. 7a). As shown in Fig. 7b and c, the temperatures of the CuFeSe_2_-AMD3100-Gem solutions rapidly increased with increasing concentration and power density. In detail, the temperature increment (ΔT) could reach 0.5 and 39.5 °C by changing their concentration (0, 12.5, 25, 50, 100 µg/mL), and ΔT was tuned between 28.5 °C and 46 °C with increasing radiation power (1.0, 1.5, 2.0 W/cm^2^). Then, the stability of the photothermal conversion was further evaluated by monitoring the temperature change in CuFeSe_2_-AMD3100-Gem solutions with 100 µg/mL for four continuous heating‒cooling cycles (Fig. 7d). The temperature evolution profile during each irradiation cycle showed no obvious change, implying the excellent photothermal stability of CuFeSe_2_-AMD3100-Gem.

**Fig. 8:**
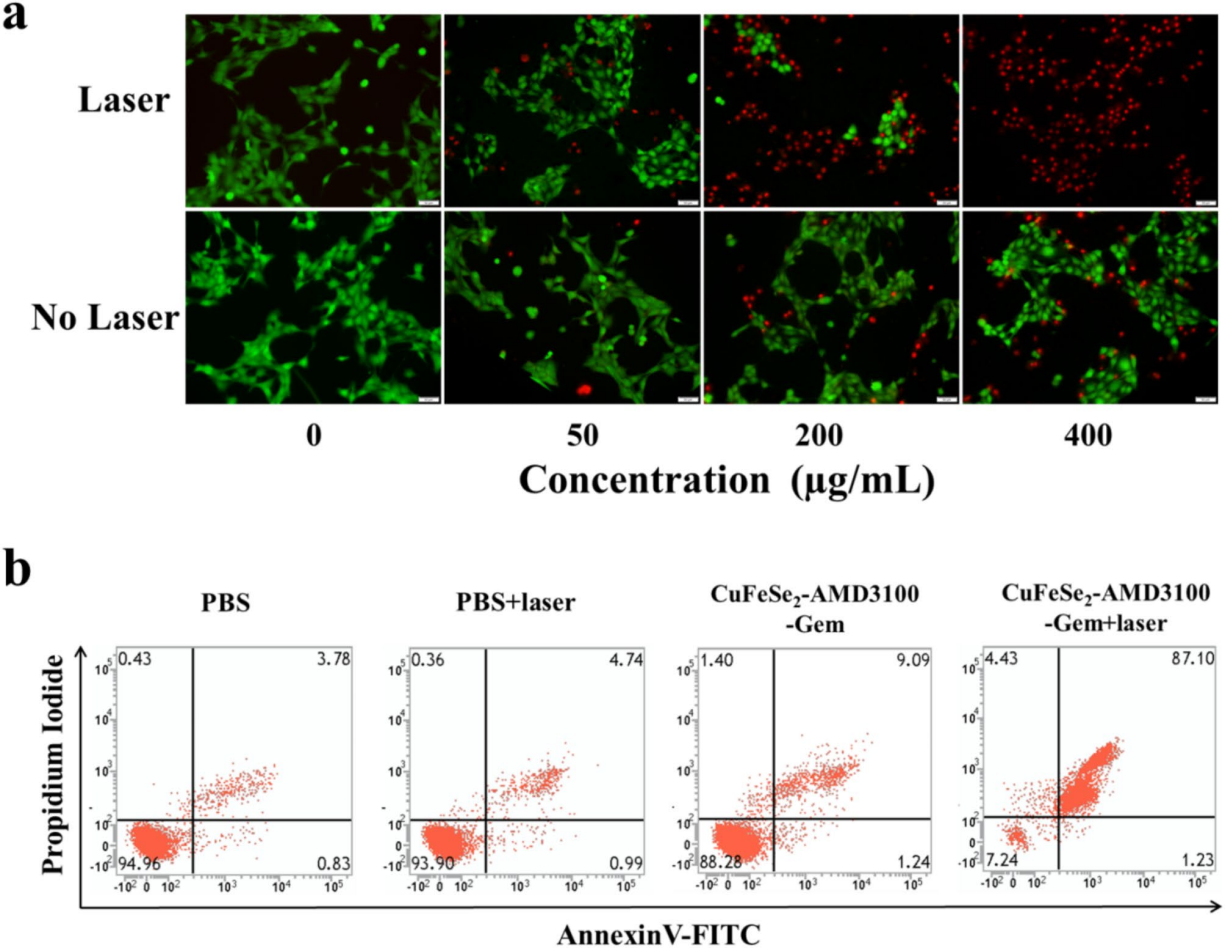
(**a**) The fluorescence images of living and dead cells detected by Calcein-AM (green, live cells) and PI (red, dead cells) double staining. Scale bar = 50 μm. (**b**) Flow cytometry analysis was performed on 4T1 cells after coincubation for 4 h

Next, live/dead analysis in vitro was performed to observe the cell death induced by the synergistic photothermal/catalysis/chemical ablation of the CuFeSe_2_-AMD3100-Gem nanosheets. The results revealed that a portion of the 4T1 cells were killed after treatment with CuFeSe_2_-AMD3100-Gem upon 808 nm laser irradiation, and the number of dead cells clearly increased with increasing CuFeSe_2_-AMD3100-Gem concentration (Fig. 8a). Comparatively, only a few dead cells were found in the no-laser group (CuFeSe_2_-AMD3100-Gem + no laser). Additionally, the anticancer effect in.

vitro was investigated via a CCK-8 assay. Various concentrations (0, 50, 100, 200, and 400 µg/mL) of CuFeSe_2_-AMD3100-Gem were used to culture 4T1 cells. It was found that 4T1 cell viability reduced with increasing concentration and that CuFeSe_2_-AMD3100-Gem exhibited some toxicity at higher concentrations (≥ 100 µg/mL), with cell viability lower than 80%, indicating the definite chemical toxicity of these nanosheets (Fig. S9). In particular, a much greater decrease in cell viability was observed upon laser exposure, further illuminating the synergistically strengthened anticancer effect. These results demonstrated that CuFeSe_2_-AMD3100-Gem effectively mediated the photothermal and chemical destruction of 4T1 cancer cells.

Furthermore, a flow cytometer was used to measure the percentage of apoptotic cells. As shown in Fig. 8b, the apoptosis rate was 10.33% in the CuFeSe_2_-AMD3100-Gem group, which was slightly greater than that in the PBS group (4.61%) and the PBS + laser group (5.73%), whereas the apoptosis rate was 88.33% in the CuFeSe_2_-AMD3100-Gem + laser group, which was significantly greater than that in the above three groups. These findings suggested that CuFeSe_2_-AMD3100-Gem nanosheets could produce a small number of apoptotic cells because of enzyme activity and chemotherapeutic effects, but the synergistic photothermal/catalytic/chemical effects mediated by CuFeSe_2_-AMD3100-Gem after laser irradiation could cause obvious apoptosis.

**Fig. 9:**
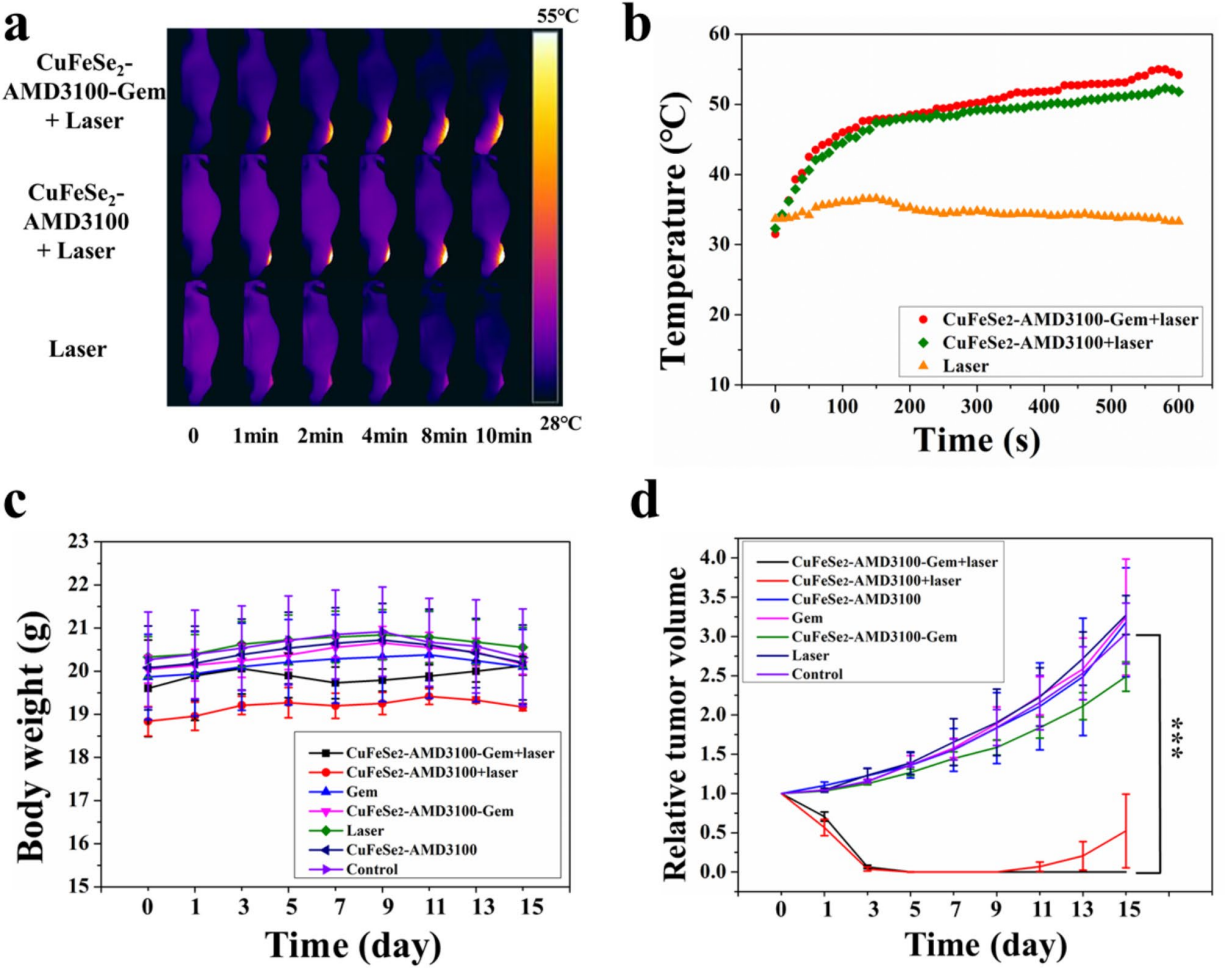
(**a**) Infrared thermal images of mice intravenously injected with CuFeSe_2_-AMD3100-Gem nanosheets and irradiated at different time intervals. (**b**) Real-time temperature change curves of the nude mice irradiated by laser-on for 10 min. (**c**) Body weights of the nude mice versus a survival time. (**d**) Relative tumor volumes of the nude mice versus a survival time

**Fig. 10:**
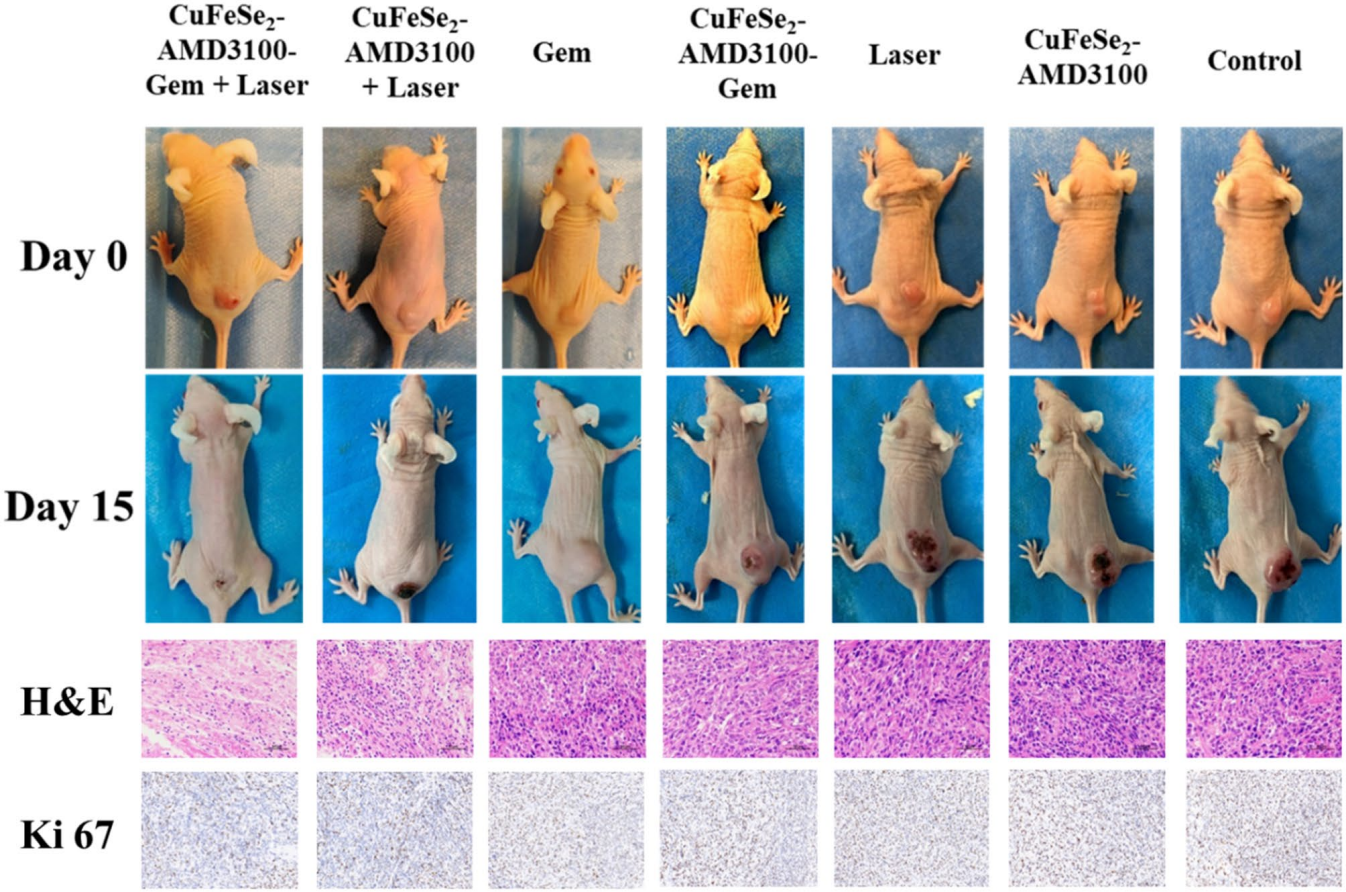
Representative photos of nude mice in different groups after PTT for 15 days and H&E, anti-Ki67 antibody staining of tumor from different groups at 24 h

### In vivo photothermal/catalytic/chemical synergistic therapeutic effect

Encouraged by the superior photothermal properties of CuFeSe_2_-AMD3100-Gem, in vivo experiments were further implemented to evaluate the synergistic photothermal/catalytic/chemical therapeutic effects on 4T1 tumors. As shown in Fig. 9a and b, the tumor site temperatures increased rapidly from 31.5 °C to 54.2 °C (ΔT = 22.7 °C) and 51.8 °C (ΔT = 20.3 °C), respectively, in the CuFeSe_2_-AMD3100-Gem + laser and CuFeSe_2_-AMD3100 + laser groups, indicating the efficient photothermal toxicity of CuFeSe_2_-AMD3100-Gem in vivo. Nevertheless, the temperature of the tumors in the laser group slightly increased to 36.6 °C during the first 150 s and then gradually decreased to 33.3 °C during the remaining time.

During the following days after laser irradiation, all nude mice were photographed and weighed, and the tumor sizes were measured every other day. Figure 9c shows that the body weights of almost all nude mice slightly increased during the first week and subsequently, decreased mildly with increasing tumor growth. Except for the CuFeSe_2_-AMD3100-Gem + laser and CuFeSe_2_-AMD3100 + laser groups, the tumors in the other groups grew rapidly, demonstrating that neither laser irradiation alone nor CuFeSe_2_-AMD3100-Gem/CuFeSe_2_-AMD3100 exposure alone inhibited tumor growth (Fig. 9d). Notably, tumors in nude mice treated with CuFeSe_2_-AMD3100-Gem + laser were thoroughly eliminated posttreatment. In addition, tumor growth was apparently inhibited in the CuFeSe_2_-AMD3100 + laser group, but unfortunately, some tumor recurrence or incomplete ablation was observed after 15 days of treatment, further demonstrating the outstanding synergistic photothermal/catalytic/chemical therapeutic effect of these nanosheets.

Furthermore, histological analysis of tumors from different groups at 24 h post treatment was carried out via H&E and anti-Ki67 antibody staining (Fig. 10). H&E staining revealed severe tumor necrosis, such as loss of nuclei and cell shrinkage, in the CuFeSe_2_-AMD3100-Gem + laser and CuFeSe_2_-AMD3100 + laser groups, whereas no obvious cell death or damage was detected in the other groups. Similarly, Ki67 antibody staining revealed less cell proliferation and potent tumor inhibition in the above two groups than in the other groups.

## Discussion

In the past decade, near-infrared (NIR)-light-triggered PTT has attracted great interest because of its high efficiency, easy operation and weak side effects. Photothermal conversion materials, which convert NIR light to heat, have the advantages of high photothermal conversion efficiency, strong photothermal stability and easy synthesis, and few also serve as CT or MRI contrast agents. Currently, some bimetallic chalcogenide nanomaterials have been reported to serve as theranostic platforms for multimodal imaging and PTT of cancer [38, 50, 51, 52]. More significantly, nanozymes have also attracted widespread attention because of their simple preparation and superior properties, which made them ideal candidates for efficient enzymatic therapy [30, 53]. Considering the superior properties of bimetallic chalcogenide materials and the limitations of single therapy, a multifunctional CuFeSe_2_-AMD3100-Gem nanosheet was successfully synthesized in this study, which could enhance the antitumor effect via the synergism of PTT, enzymatic therapy and chemotherapy. In vitro and in vivo experiments confirmed that these nanosheets may be an efficient platform for MRI/CT dual-modality imaging and photothermal/catalytic/chemical synergistic therapy for tumors.

In the biomedical field, 2D nanomaterials provide a new opportunity for cancer diagnosis and treatment because of their excellent physicochemical properties, e.g., high photothermal conversion efficiency and high uploading of drugs. To date, various types of 2D nanomaterials have been developed and attracted the investigators’ attention, in which 2D ternary chalcogenides have been reported for imaging and therapy of cancer, such as CuFeS_2_ nanoplates [16], CFS@DOX nanosheets [39], CFS-S-DOX nanosheets [40]. Inspired by the extraordinary advantages of these 2D nanomaterials, a multifunctional theranostic nanosheet was developed based on Cu-Fe-Se that integrates PTT, catalytic therapy and Gem-based chemotherapy to improve therapeutic efficiency, which represents an innovation in our design. In recent years, the resulting nanostructures based on Cu-Fe-Se, including BG-CFS [37], CuFeSe_2_@MIL-100(Fe)-AIPH [41], CuFeSe_2_-PEG-FA [36], MPEG-PCL@CuFeSe_2_ [43], CuFeSe_2_-AIPH@BSA [42], and CuFeSe_2_@PTMP − PMAA [38], have been used as contrast agents for MRI and photosensitizers for PTT because of their induced magnetism and enhanced photothermal conversion properties. However, compared with these spherical nanoparticles, there have been only a few reports on 2D Cu-Fe-Se nanostructures for biomedical applications. For example, functionalized CFS@DOX nanosheets were used for CT imaging and single-photon emission computed tomography (SPECT) imaging after they were labeled with radioactive ^99m^Tc, which simultaneously exhibited strong antitumor efficacy in combination with photothermal therapy [39]. Doxorubicin-loaded CFS-S nanosheets have also been proven to be promising agents for achieving synergistic therapeutic effects [40]. Hence, given the advantages of 2D Cu-Fe-Se nanostructures, it is highly important to prepare biocompatible high-performance nanosheets for combined imaging and therapy.

In this study, CuFeSe_2_-AMD3100-Gem nanosheets were designed as effective theranostic agents for MRI/CT/Infrared thermal imaging and photothermal/catalytic/chemical combined therapy. It was found that CuFeSe_2_-AMD3100-Gem exhibited outstanding POD-like activites, which could convert endogenous H_2_O_2_ into hydroxyl radicals (•OH) for catalytic therapy, and their enzyme-like activities were obviously enhanced under light irradiation. Surprisingly, only a few studies have reported the enzyme-like activity of Cu-Fe-Se materials for tumor theranostics. Therefore, in our study, the POD-like activity of CuFeSe_2_-AMD3100-Gem was utilized in combination with hyperthermia killing and chemical damage to achieve favorable synergistic anticancer efficacy. In addition, in vitro and in vivo experiments verified the good dispersion, excellent biosafety and superior targeting specificity of the CuFeSe_2_-AMD3100-Gem nanosheets, demonstrating their potential for in vivo biological application. Currently, CT and MRI techniques are crucial for the diagnosis and treatment of patients with breast cancer, but the use of contrast agents improves the sensitivity and accuracy of in vivo imaging. In this study, the MRI results revealed that the CuFeSe_2_-AMD3100-Gem solution displayed a strong T2 enhancement effect with increasing concentration, and a significant T2 blackening effect was simultaneously observed in the tumor region after the administration of the nanosheets. Furthermore, the CT value of CuFeSe_2_-AMD3100-Gem was greater than that of clinically approved iohexol at the same concentration according to in vitro and in vivo evaluations. These results indicated that CuFeSe_2_-AMD3100-Gem had superior contrast efficacy for MRI and CT imaging. To date, some nanomaterials (e.g. I@CNDs-Fe_3_O_4_, BiVO_4_/Fe_3_O_4_@PDA, HA-FeWO_4_ and other CuFeSe_2_-based nanomaterials) have been designed as bimodal contrast agents for CT and T2 weighted imaging in vitro or in vivo [54–57]. These nanostructures exhibited superior magnetic properties with a transverse relativity (r_2_) ranging from 4.2809 mM^− 1^s^− 1^ to 186.7428 mM^− 1^s^− 1^, indicating the great promise of these nanomaterials as T2-weighted MRI contrast agents. Compared with these nanomaterials, the T2 signal of prepared CuFeSe_2_-AMD3100-Gem nanosheets showed a strong concentration-dependent enhancement effect with an r_2_ value of 6.6553 mM^− 1^s^− 1^, suggesting a moderate MR enhancement performance of these nanosheets. Notably, in most studies, an intratumoral injection was performed to achieve T2 enhancement effect of the tumor region in vivo while the tumor region showed a remarkable darkening effect after intravenous injection of these nanosheets in our study, highlighting the superior magnetism and clinical applicability of CuFeSe_2_-AMD3100-Gem nanosheets. Overall, CuFeSe_2_-AMD3100-Gem could serve as a promising theranostic agent for combined imaging and therapy.

Nevertheless, there are still some limitations to this study. First, simple nude mouse models were used in our study rather than some mammals and primates, as they may better mimic human disease. Furthermore, only 4T1 tumors were used to validate the MRI/CT imaging capability and anticancer efficacy of CuFeSe_2_-AMD3100-Gem. Hence, imaging and therapeutic evaluation of other diseases will be the next goal. In addition, considering the excellent properties of ternary chalcogenides, other new agents based on CuFeSe_2_ will be developed for further biomedical research. More importantly, it is necessary to further explore the mechanism of tumor cell killing induced by these nanomaterials.

## Conclusion

In conclusion, novel CuFeSe_2_ ternary nanozymes for image-guided photothermal/catalytic/chemical synergistic therapy were successfully developed. The CuFeSe_2_ ternary nanozymes exhibit good colloidal stability, superior biocompatibility, high photothermal conversion efficiency, and excellent targeting ability, suggesting it promises as nanotheranostic agent for in vivo applications. Furthermore, their unique structure and targeting specificity make them ideally suitable for MRI and CT dual-modality imaging. More importantly, CuFeSe_2_ ternary nanozymes successfully kill cancer cells through cell ablation induced by combined photothermal/catalytic/chemical effects due to their strong photothermal conversion efficiency and enzyme-like activities, further highlighting their potential in the precise diagnosis and treatment of TNBC. The present findings indicate that CuFeSe_2_ ternary nanozymes may be a promising candidate for the theranostics of TNBC in the future.

## Electronic supplementary material

Below is the link to the electronic supplementary material.

Supplementary Material 1

## Data Availability

Data will be made available on request.
