# Supplementary material for: Assessment of CuFeSe2 ternary nanozymes for multimodal triple negative breast cancer theranostics
Source: Nano Converg. 2025 Apr 2;12:18. doi: 10.1186/s40580-025-00483-4 (PMC11965050; doi:10.1186/s40580-025-00483-4)

**Supporting Information**

**Assessment of CuFeSe_2_ ternary nanozymes for multimodal triple negative breast cancer theranostics**

Chunmei Yang^1†^, Lihong Li^1^^†^, Mingdong Li^1^^†^, Yue Shu^2^, Yiping Luo^3^, Didi Gu^1^, Xin Zhu^1^, Jing Chen^1*^, Lu Yang^1^^, 3*^, Jian Shu^1*^

^1^ Department of Radiology, The Affiliated Hospital of Southwest Medical University and Precision Imaging and Intelligent Analysis Key Laboratory of Luzhou, Luzhou, Sichuan, China, 646000.

^2^ Department of Oncology, The Affiliated Hospital of Southwest Medical University, Luzhou, Sichuan, China, 646000.

^3^ The Affiliated Hospital, Southwest Medical University, Luzhou, 646000, China

^†^ These authors contributed equally to this work.

^*^ Corresponding author:

Jing Chen: E-mail: yzqdcj@126.com

Lu Yang: E-mail: yanglu@swmu.edu.cn

Jian Shu: E-mail: shujiannc@163.com

**Table of contents**

1. [**Table S1-2 2**](#_Toc508482800)

**2.** **Scheme S1-2 3**

**3. Figure S1-9 4**

**4. ^1^H NMR and ^13^C NMR Spectra 8**

# Table S1-2

**Table S1** Parameters of some sequences of in vitro MRI scanning

|  | T1WI | T2WI | T1 mapping | T2 mapping |
| --- | --- | --- | --- | --- |
| Slice thickness (mm) | 1.4 | 1.4 | 1.4 | 1.4 |
| Field of view (mm×mm) | 180×180 | 180×180 | 180×180 | 180×180 |
| Matrix | 256×256 | 256×256 | 256×256 | 256×256 |
| Repetition time (ms) | 600 | 200 | 5.62 | 2280 |
| Echo time (ms) | 7.5 | 96 | 2.67 | 12.9 |
| Number of excitations | 3 | 4 | 6 | 1 |

**Table S2** Parameters of T2WI and T2* mapping in vivo.

|  | T2WI | T2* mapping |
| --- | --- | --- |
| Slice thickness (mm) | 1.4 | 2 |
| Field of view (mm×mm) | 180×180 | 180×180 |
| Matrix | 256×256 | 256×256 |
| Repetition time (ms) | 2000 | 293 |
| Echo time (ms) | 96 | 2.98 |
| Number of excitations | 2 | 1 |

# Scheme S1-2


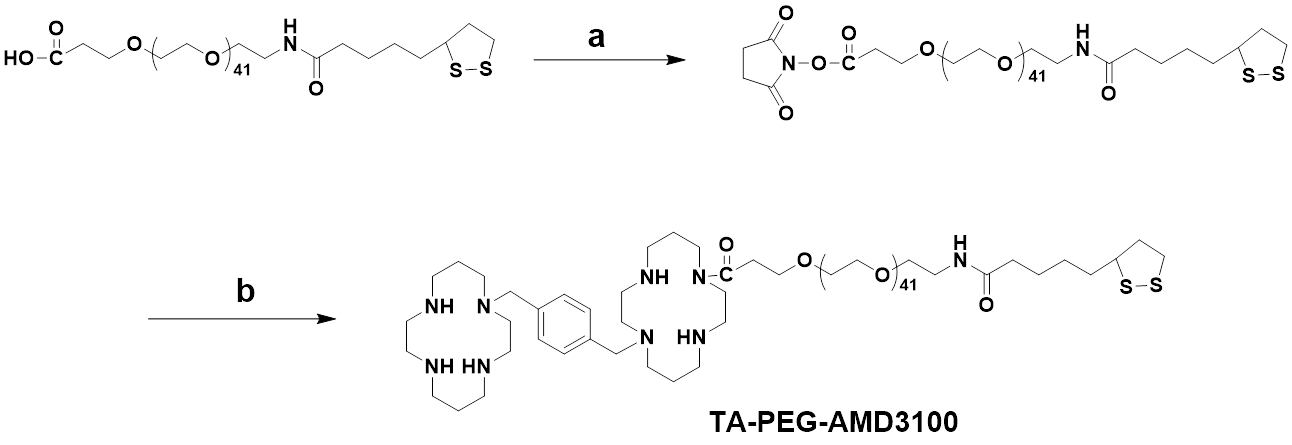


**Scheme S1**. Synthesis of TA-PEG-AMD3100. *Reagents and condition*s: (a) NHS, DCC, DCM, RT, 12 h; (b) AMD3100, NaHCO_3_, THF, H_2_O, RT, 48 h.


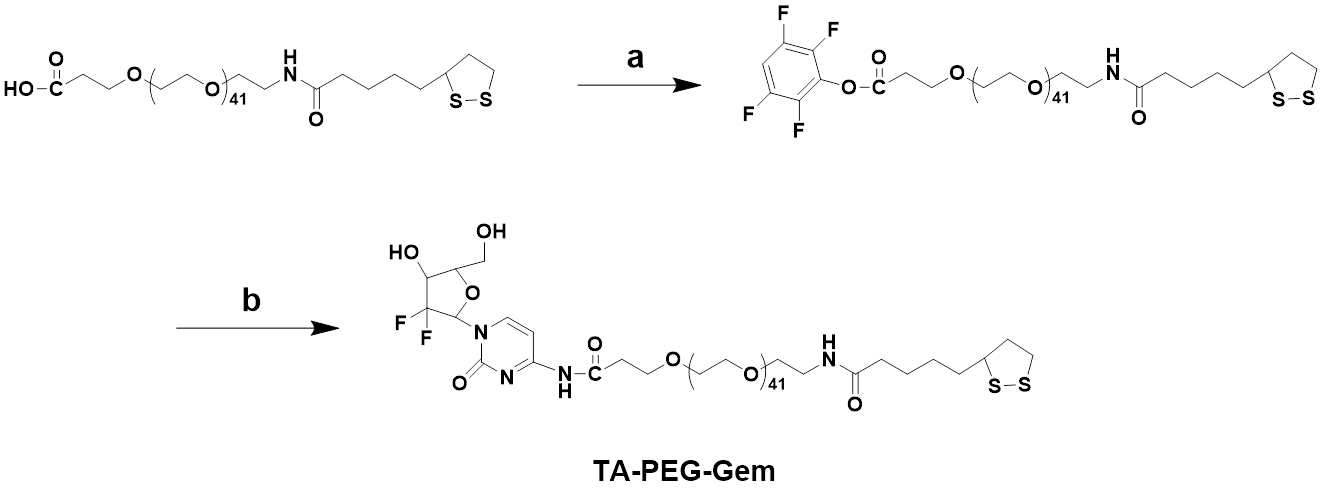


**Scheme S2.** Synthesis of TA-PEG-Gem. *Reagents and conditions*: (a) 2,3,5,6-Tetrafluorophenol, DCC, DMSO, 90 °C, 12 h; (b) Gemcitabine hydrochloride, N, N-diisopropylethylamine, DMSO, 80 °C, 12 h.

# Figure S1-9


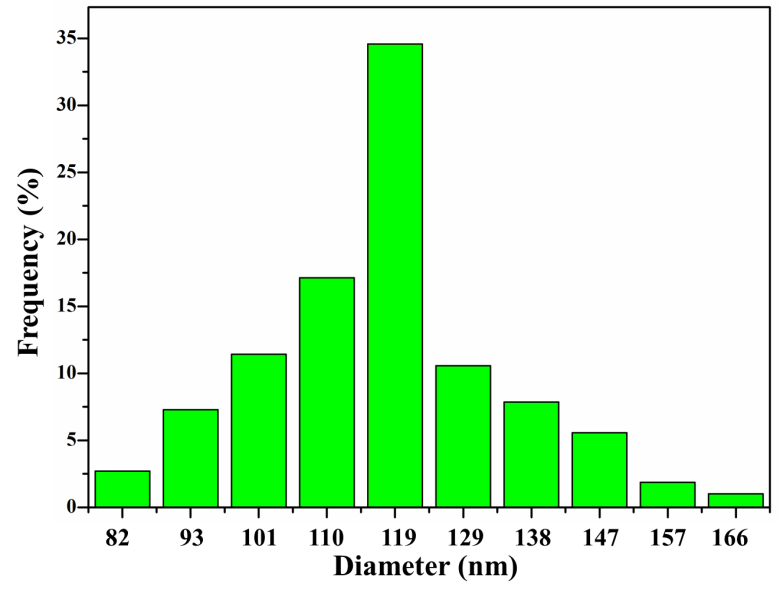


**Fig. S1** Particle size distribution histograms of CuFeSe_2_-AMD3100-Gem nanosheets, counted from 242 nanosheets shown in typical TEM images.


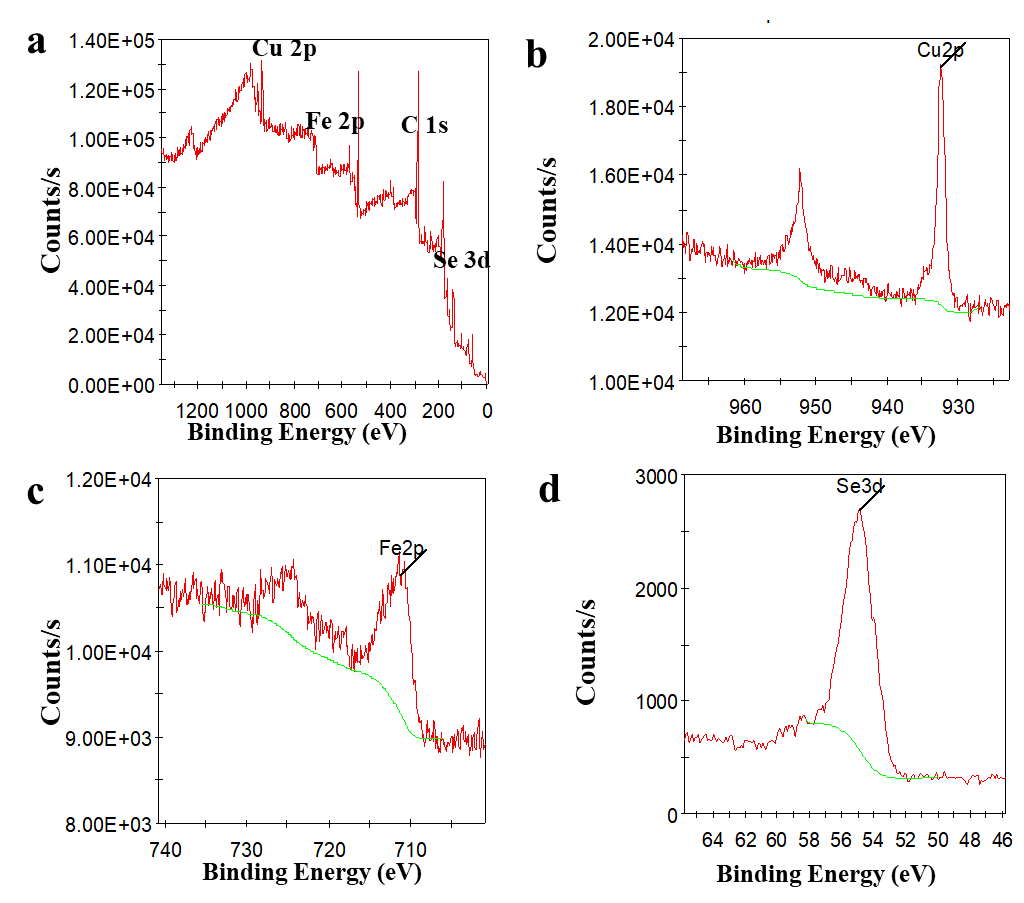


**Fig. S2**. (a) XPS spectrum of CuFeSe_2_-AMD3100-Gem nanosheets. (b-d) The high-resolution XPS spectra of Cu 2p, Fe 2p and Se 3d respectively.


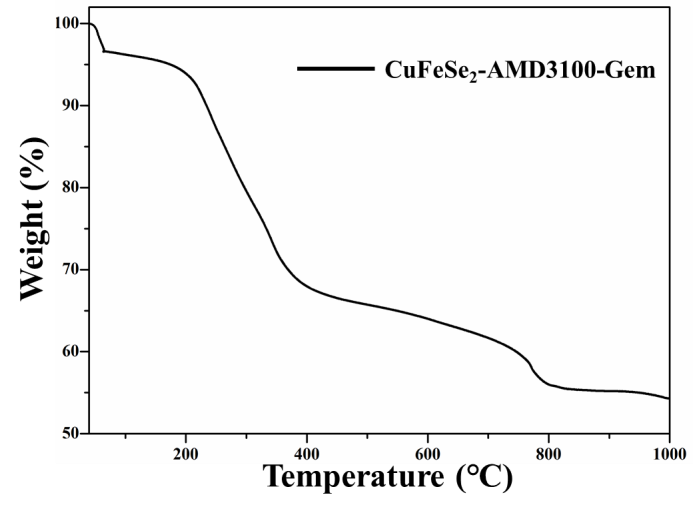


**Fig. S3** TGA curves of the CuFeSe_2_-AMD3100-Gem nanosheets.


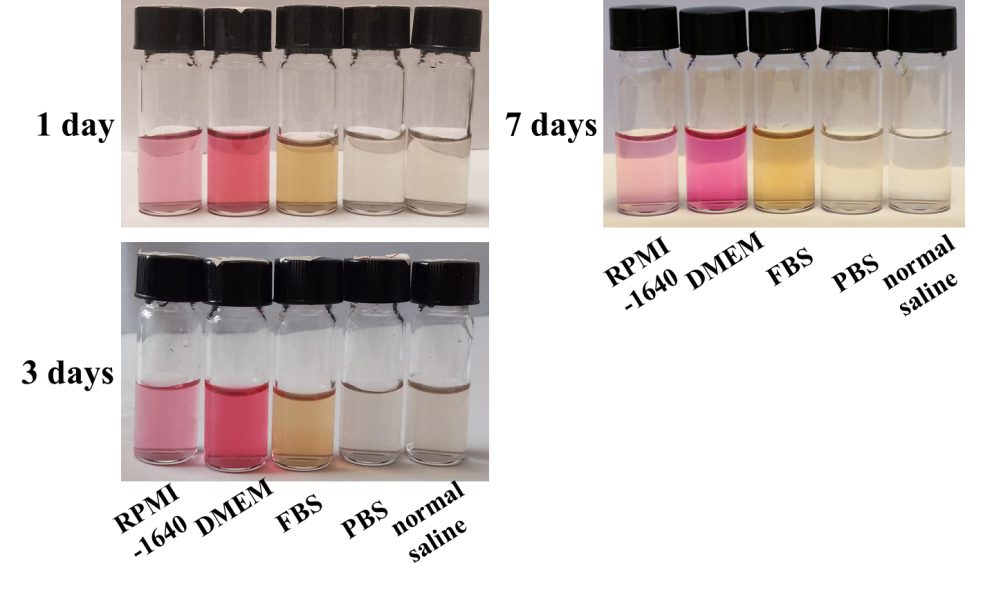


**Fig. S4** The colloidal stability of CuFeSe_2_-AMD3100-Gem nanosheets in different media (100 mg/mL, from left to right: RPMI-1640, DMEM, FBS, PBS, normal saline) at 37°C for 1,3 and 7 days.


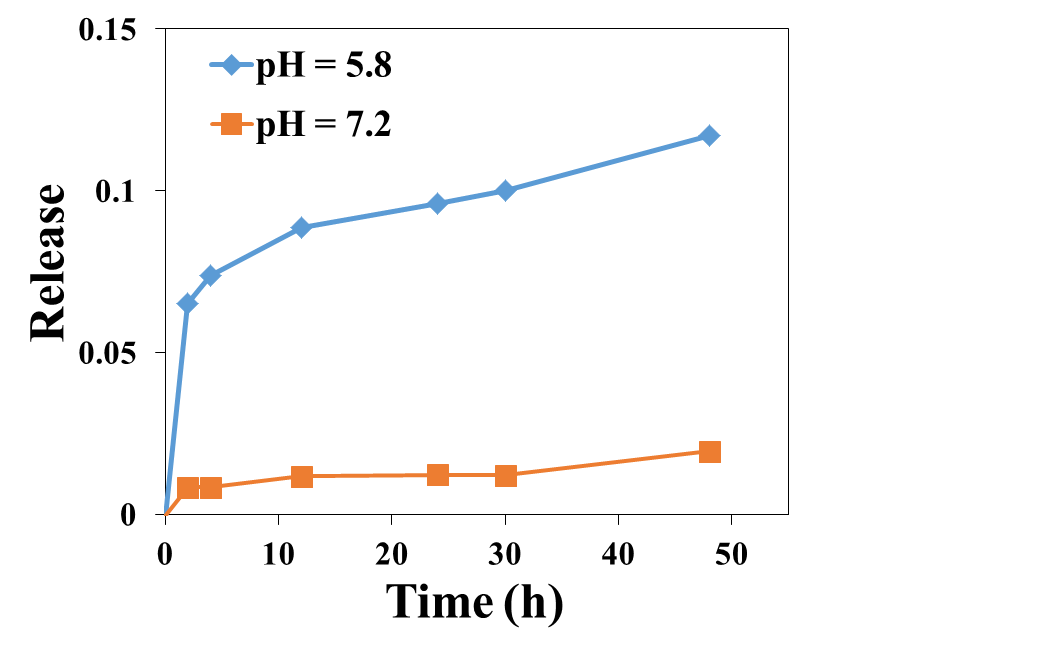


**Fig. S5** The Gem release profiles of CuFeSe_2_-AMD3100-Gem nanosheets at pH 5.8 and pH 7.2 in phosphate buffer.


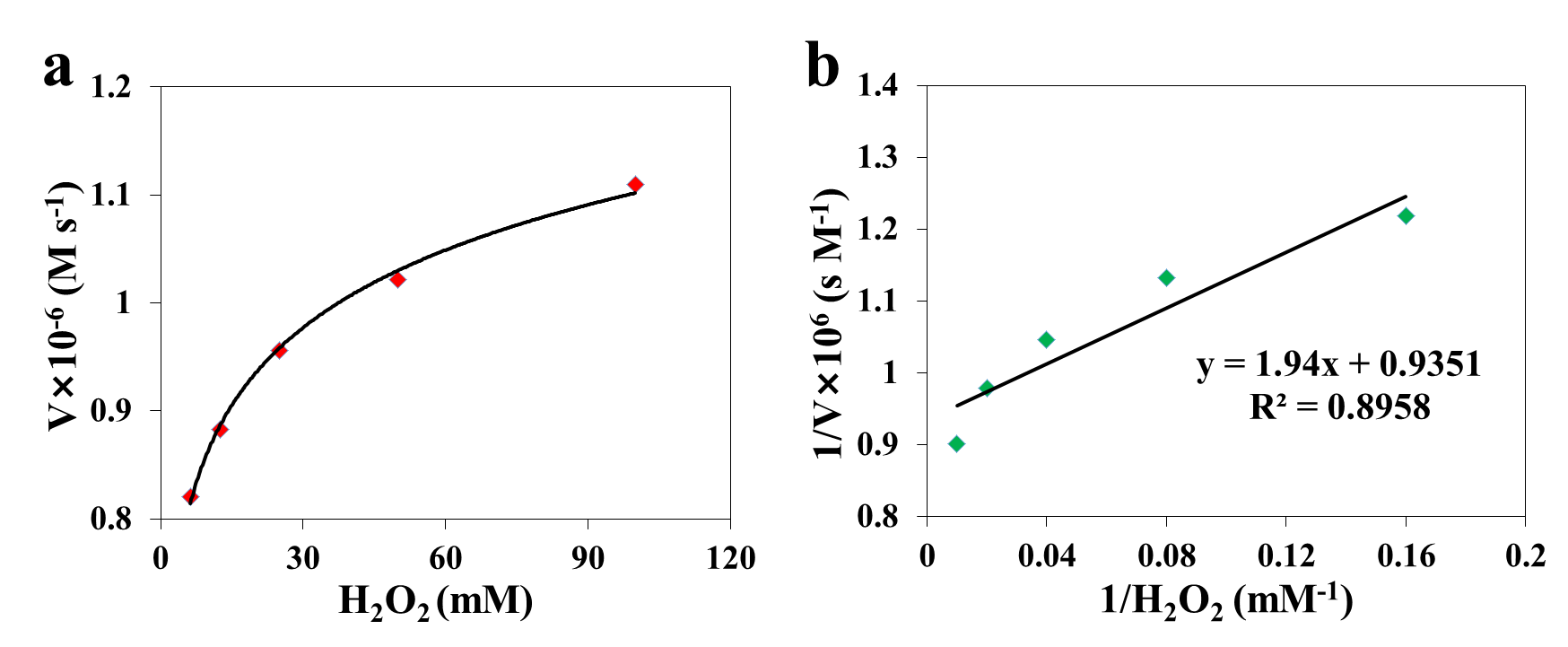


**Fig. S6** (a) Kinetics of CuFeSe_2_-AMD3100-Gem nanaosheets for H_2_O_2_ substrate. (b) Lineweaver-Burk double reciprocal plot curve.


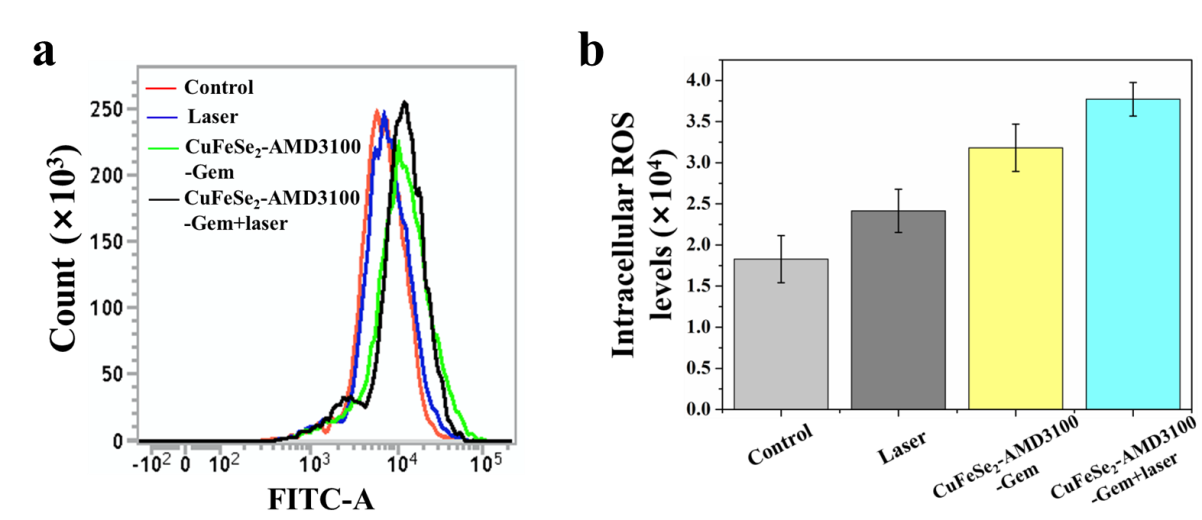


**Fig. S7** Flow cytometry analysis of intracellular ROS levels in 4T1 cells treated with control, laser, CuFeSe_2_-AMD3100-Gem, and CuFeSe_2_-AMD3100-Gem + laser respectively.


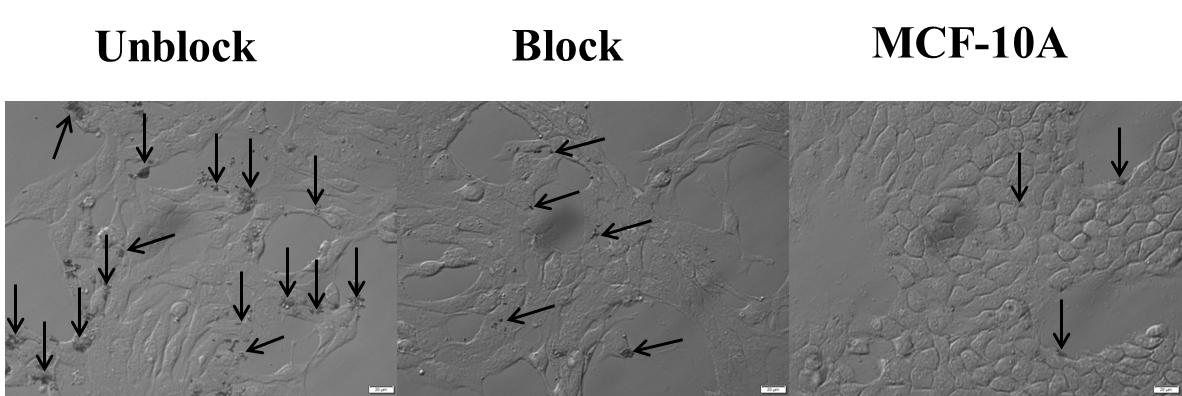


**Fig. S8** Optical microscope images of 4T1 and MCF-10A cells treated with CuFeSe_2_-AMD3100-Gem (50 μg/mL). The black arrows represent some CuFeSe_2_-AMD3100-Gem nanosheets binding to the surface of 4T1/MCF-10A cells. Scale bar: 20 μm.


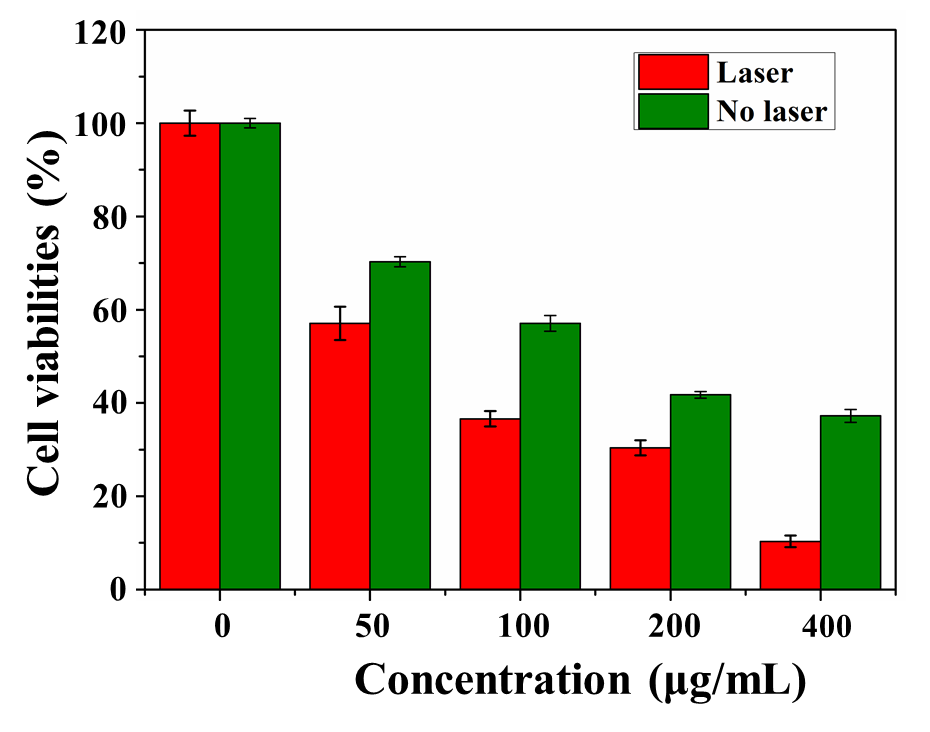


**Fig. S9** Cell viabilities of 4T1 cells determined by CCK-8 assay after incubation with different concentrations of CuFeSe_2_-AMD3100-Gem nanosheets with or without the laser treatment.

**^1^H NMR and ^13^C Spectra**

**^1^H NMR spectrum of TA-PEG-NHS**


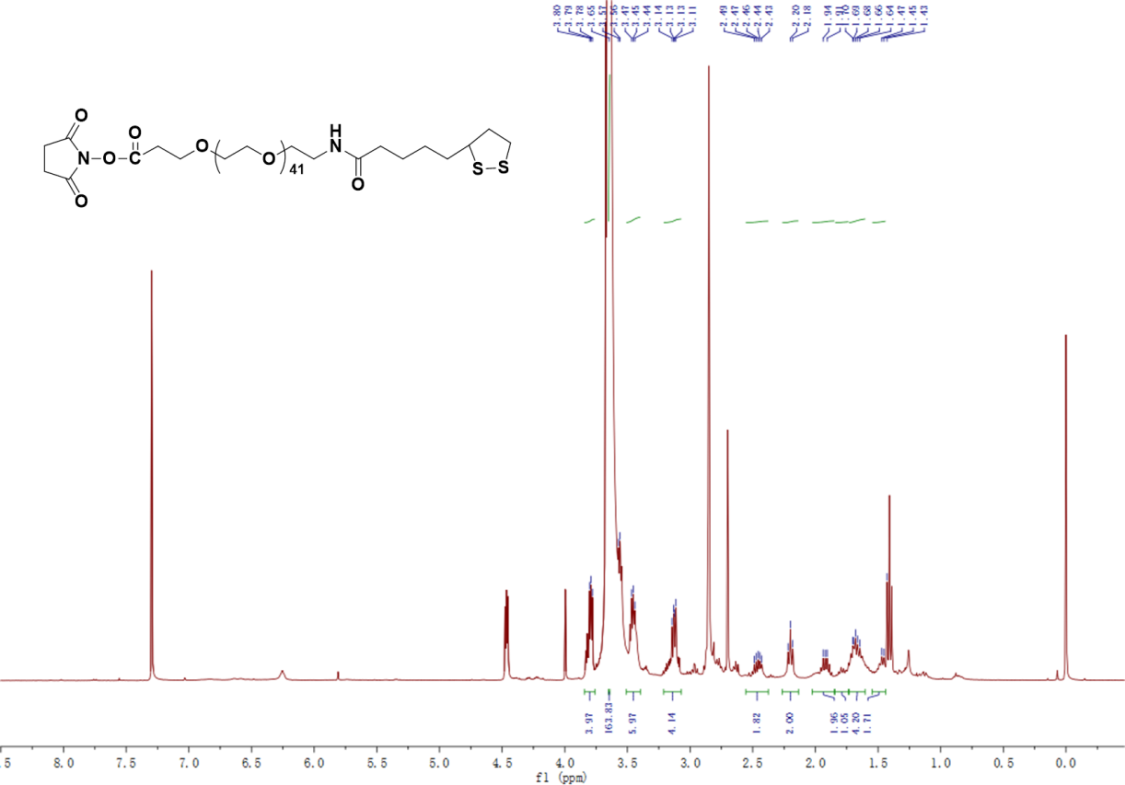


**^13^C NMR spectrum of TA-PEG-NHS**


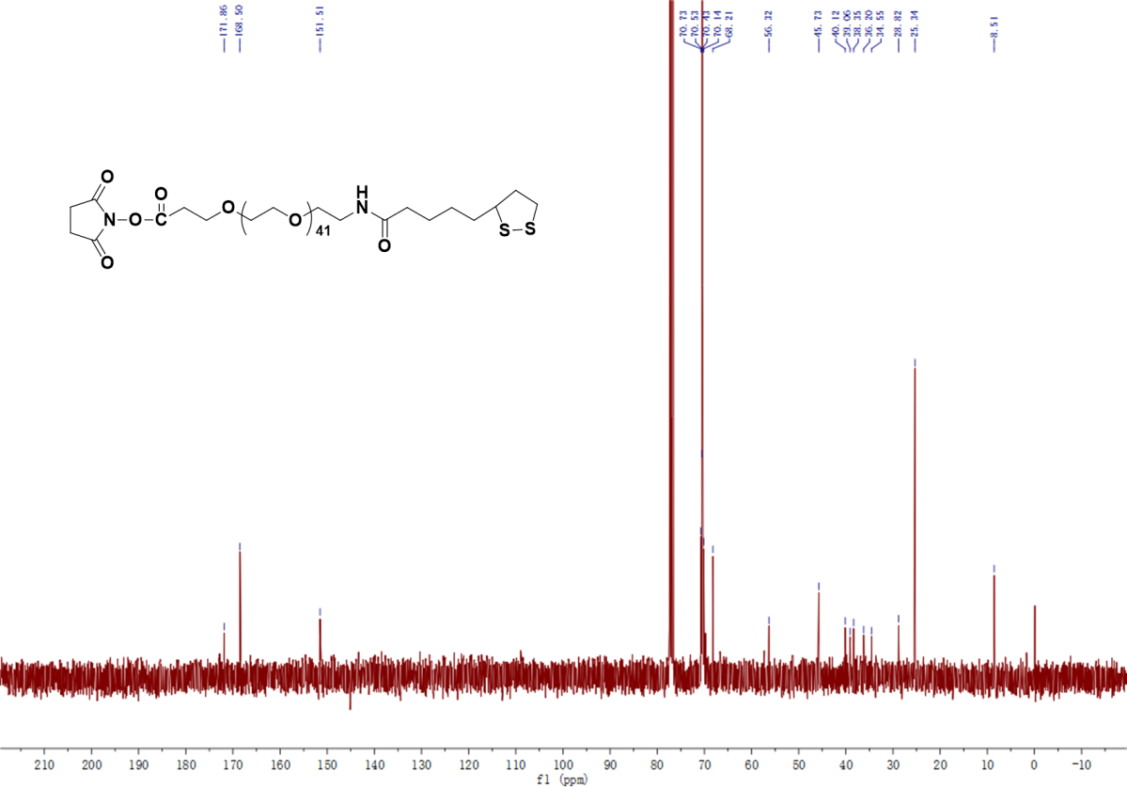


**^1^H NMR spectrum of TA-PEG-AMD3100**


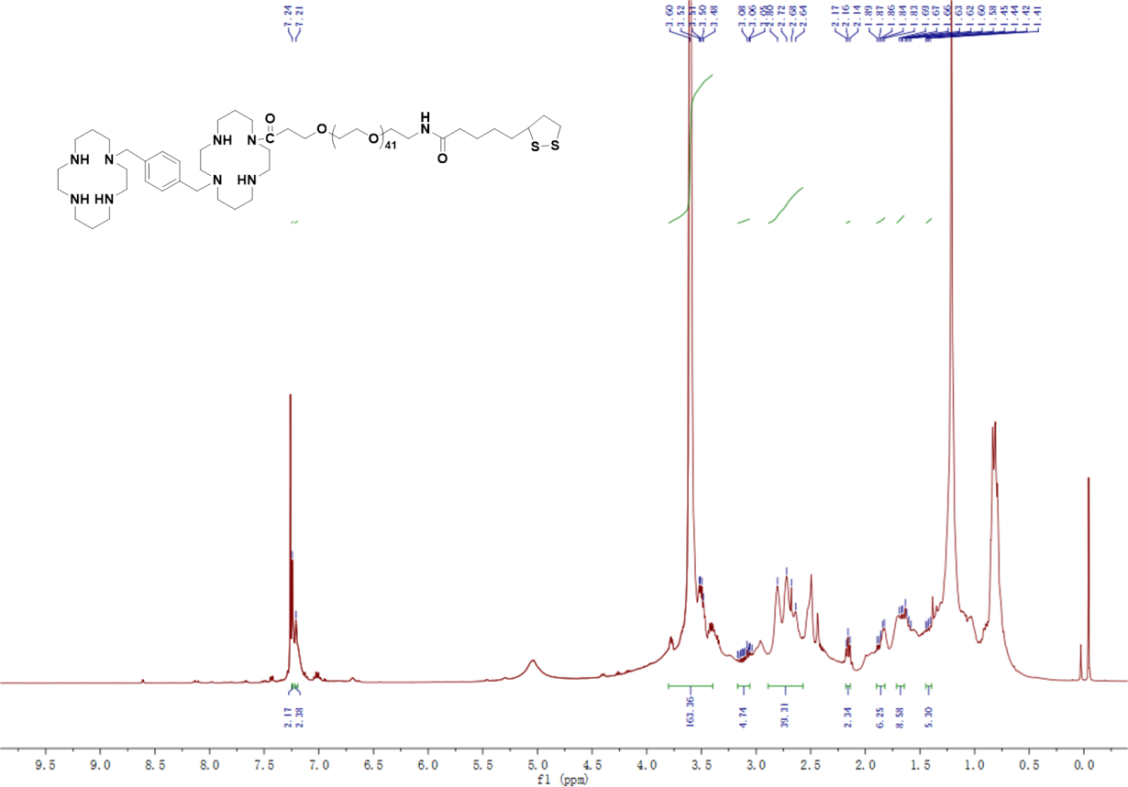


**^13^C NMR spectrum of TA-PEG-AMD3100**


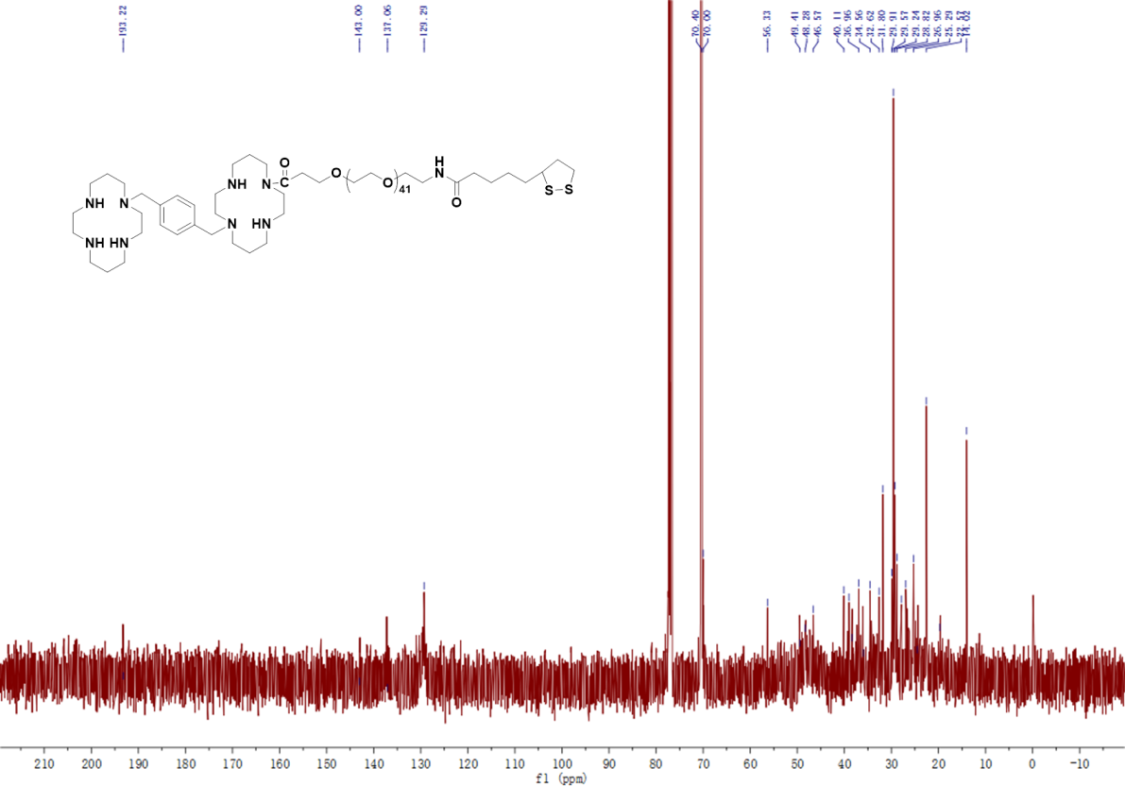


**^1^H NMR spectrum of TA-PEG-Gem**


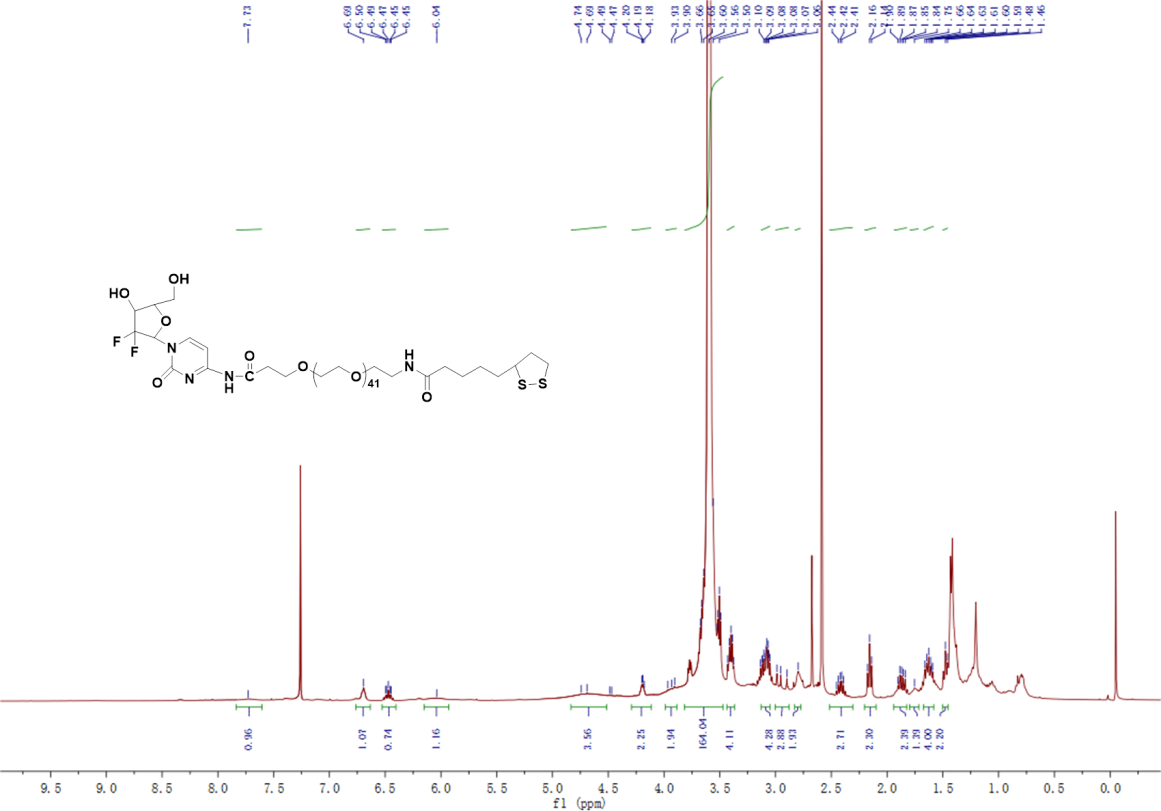


**^13^C NMR spectrum of TA-PEG-Gem**


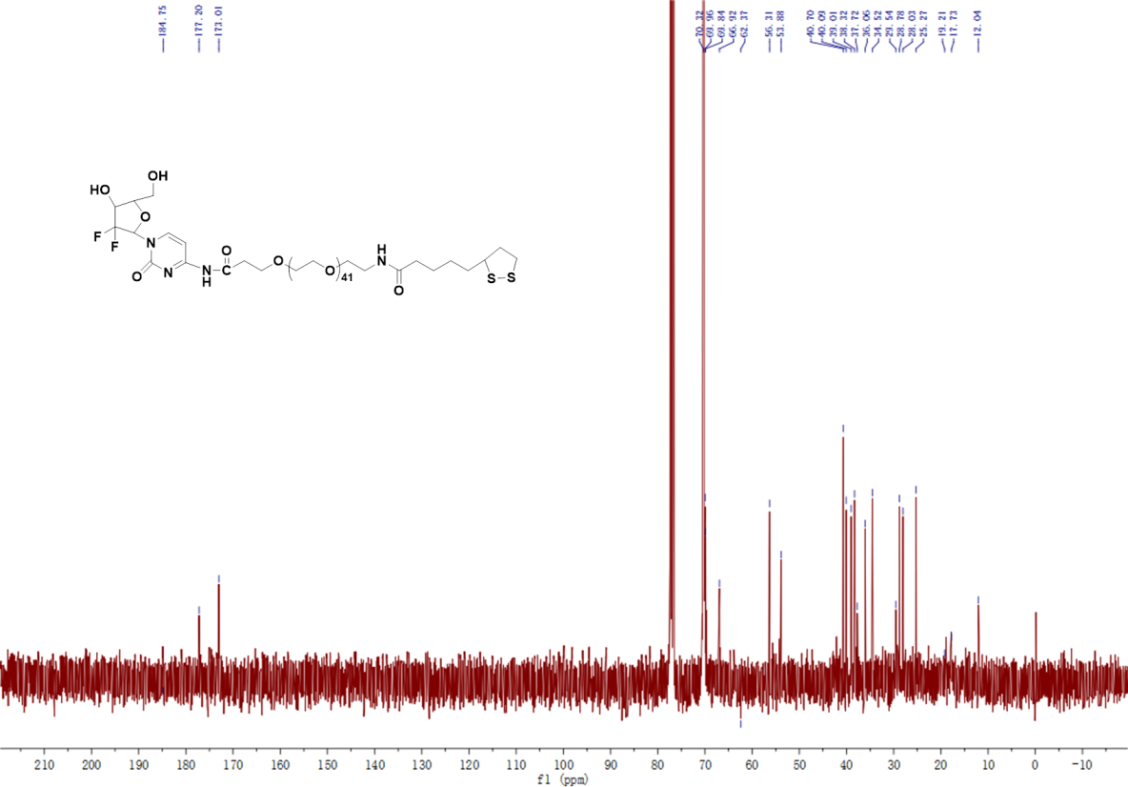

Supplement: Supplementary file 1 — Supplementary Material 1 [file 40580_2025_483_MOESM1_ESM.docx]
